# A Bioresponsive Genetically Encoded Antimicrobial Crystal for the Oral Treatment of *Helicobacter Pylori* Infection

**DOI:** 10.1002/advs.202301724

**Published:** 2023-09-07

**Authors:** Wenxiu Zhang, Zaofeng Yang, Jiale Zheng, Kaili Fu, Jack Ho Wong, Yunbi Ni, Tzi Bun Ng, Chi Hin Cho, Michael K. Chan, Marianne M. Lee

**Affiliations:** ^1^ School of Life Sciences and Center of Novel Biomaterials The Chinese University of Hong Kong Hong Kong 999077 China; ^2^ Department of Medicine and Therapeutics Faculty of Medicine The Chinese University of Hong Kong Hong Kong 999077 China; ^3^ School of Biomedical Sciences Faculty of Medicine The Chinese University of Hong Kong Hong Kong 999077 China; ^4^ Department of Anatomical and Cellular Pathology Prince of Wales Hospital The Chinese University of Hong Kong Hong Kong 999077 China; ^5^ Present address: School of Health Sciences Caritas Institute of Higher Education Hong Kong 999077 China; ^6^ Present address: School of Pharmacy University of Southwest Medical University Luzhou 646000 China

**Keywords:** antimicrobial peptide, gut microbiota, *Helicobacter pylori*, LL‐37, protein crystals

## Abstract

*Helicobacter pylori* (*H. pylori*) causes infection in the stomach and is a major factor for gastric carcinogenesis. The application of antimicrobial peptides (AMPs) as an alternative treatment to traditional antibiotics is limited by their facile degradation in the stomach, their poor penetration of the gastric mucosa, and the cost of peptide production. Here, the design and characterization of a genetically encoded *H. pylori*‐responsive microbicidal protein crystal Cry3Aa‐MIIA‐AMP‐P17 is described. This designed crystal exhibits preferential binding to *H. pylori*, and when activated, promotes the targeted release of the AMP at the *H. pylori* infection site. Significantly, when the activated Cry3Aa‐MIIA‐AMP‐P17 crystals are orally delivered to infected mice, the Cry3Aa crystal framework protects its cargo AMP against degradation, resulting in enhanced in vivo efficacy against *H. pylori* infection. Notably, in contrast to antibiotics, treatment with the activated crystals results in minimal perturbation of the mouse gut microbiota. These results demonstrate that engineered Cry3Aa crystals can serve as an effective platform for the oral delivery of therapeutic peptides to treat gastrointestinal diseases.

## Graphical abstract

A genetically encoded antimicrobial crystal equipped with a Ca^2+^/pH‐induced linker and a targeting peptide is used to deliver the antimicrobial peptide LL‐37 into mouse stomach via oral administration. LL‐37 is released at the site of infection in response to the elevated pH mediated by *H. pylori*‐secreted urease. The targeted killing of *H. pylori* leaves the gut microbes untouched and restores gut health.

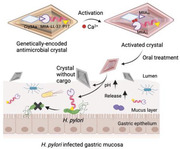

## 1 Introduction

*Helicobacter pylori* (*H. pylori*) is a human gastric pathogen that colonizes more than half of the world's population.^[^ ^1^ ^]^ It is an etiological factor for multiple gut diseases including chronic active gastritis, duodenal or gastric ulcers, and gastric mucosa‐associated lymphoid‐tissue (MALT) lymphoma,^[^ ^2^ ^]^ and has been classified as “group 1 (definite carcinogen)” based on epidemiologic studies linking the bacterium to carcinogenesis.^[^ ^3^ ^]^ *H. pylori* infection has shown to alter gut microbiota^[^ ^4^ ^]^ and causes dysbiosis, which has been implicated in the development of various diseases, including cardiovascular disease,^[^ ^5^ ^]^ colorectal cancer,^[^ ^6^ ^]^ and Alzheimer's disease.^[^ ^7^ ^]^ Current treatments for *H. pylori* infections are primarily based on a combination (dual/triple/quadruple) therapy consisting of a proton‐pump inhibitor (PPI) and/or bismuth, metronidazole, clarithromycin, amoxicillin.^[^ ^8^ ^]^ However, the increasing number of cases with poor clinical outcomes^[^ ^9^ ^]^ due to drug resistance has become a major concern. Furthermore, the negative impact of antibiotics on the gut microbiota compound the problem given the crucial role of the gut microbiome in the maintenance of human health.^[^ ^10^ ^]^ Thus, the optimal treatment for *H. pylori* infection should not only be effective for the eradication of *H. pylori*, but also help to restore the gut microbiome to a healthy state. A promising alternative to these antibiotic therapies is antimicrobial peptides (AMPs) that form the first line of host defense against invading bacteria, fungi, and viruses.^[^ ^11^ ^]^ Among them, cathelicidins and defensins are two main families of AMPs critical to the innate and adaptive immunity in mammals,^[^ ^12^ ^]^ and which have been reported to possess anti‐*H. pylori* activity^[^ ^13^ ^]^ and maintain microbiome homeostasis.^[^ ^14^ ^]^

Despite their potential as therapeutic agents, relatively few AMPs have successfully advanced through clinical trials.^[^ ^15^ ^]^ One major hurdle is their susceptibility to proteolytic degradation,^[^ ^16^ ^]^ an issue that is even more challenging for AMPs targeting *H. pylori* due to the acidic and proteolytic environment in the stomach where these pathogens reside. Approaches such as cyclization, thiolation or amidation of the N‐ or C‐terminus, pegylation, and the use of unnatural amino acids have been explored as strategies to protect the AMP against degradation.^[^ ^17^ ^]^ However, the high cost of synthesis and potentially reduced activity of the modified peptides limit the utility of these approaches. Thus, a platform that could facilitate the efficient production of the AMP and its protection against proteolytic degradation is highly desirable.

Our laboratory has developed a distinct delivery system based on sub‐micrometer‐sized Cry3Aa protein crystals that naturally form within the bacterium *Bacillus thuringiensis* (*Bt*). Cry3Aa has been used for several decades as biopesticides and is found to be harmless to humans and other mammals.^[^ ^18^ ^]^ In fact, its gene has been incorporated into many food crops for their pest resistance.^[^ ^19^ ^]^ We thus hypothesized that Cry3Aa protein crystals should exert minimal toxicity to humans as a therapeutic carrier. Indeed, over the course of developing Cry3Aa crystals as a protein/peptide delivery platform, we have found that Cry3Aa crystals exhibit no cytotoxicity to mammalian cells at therapeutic concentrations.^[^ ^20^ ^]^ Moreover, they possess numerous attributes that are advantageous for therapeutic delivery. For instance, Cry3Aa was highly amenable to genetic modification as demonstrated by its ability to accommodate different fusion proteins and still formed crystals in the *Bt* cells.^[^ ^20^, ^21^ ^]^ More importantly, the Cry3Aa crystal framework was shown to retain the function of the cargo protein and confer stability and protection to its encapsulated cargo from proteases, thereby prolonging its in vitro and in vivo lifetimes.^[^ ^20^, ^21^ ^]^ This is best illustrated by our recent study showing that the Cry3Aa crystal‐mediated delivery of the antileishmanial peptide dermaseptin S1 (DS1) led to the dramatic enhancement of its IC_50_ and therapeutic index by >30‐ and >80‐fold respectively compared with the free DS1 peptide.^[^ ^20^ ^]^ Previous studies have demonstrated that Cry3Aa crystals were stable in acidic pH.^[^ ^21a^ ^]^ We therefore surmised that it could be a well‐suited vehicle to facilitate the delivery of effective concentrations of AMP to sites of *H. pylori* infections in the stomach, thereby enhancing the therapeutic efficacy of the AMP, which in turn should enable the reduction of the dose levels required for treatment and help minimizing side effects.

**Scheme 1:**
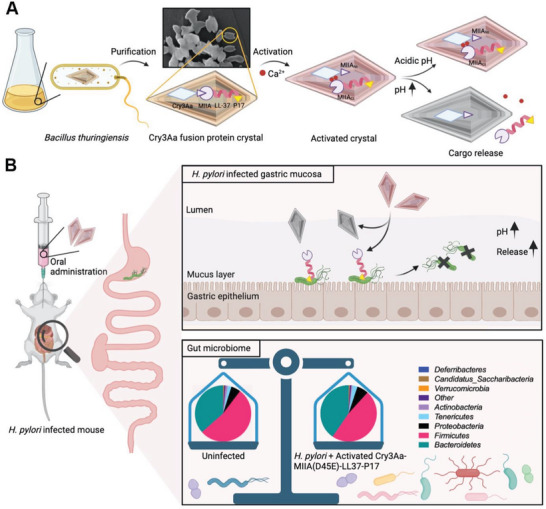
Conceptual blueprint of the bioresponsive Cry3Aa‐MIIA(D45E)‐LL37‐P17 crystal for the targeted killing of *H. pylori*. A) Cry3Aa‐MIIA(D45E)‐LL37‐P17 is endogenously synthesized as fusion protein crystals in *Bt* and can be easily purified by sucrose gradient centrifugation. Ca^2+^ activation of the purified crystals triggers the cleavage of the MIIA‐linker. The cargo LL‐37 remained encapsulated in the crystals during transit through the acidic GI tract. Upon encountering *H. pylori*, the LL‐37 is liberated from the cleaved crystals due to the pH elevation around *H. pylori* mediated by urease. Inset is a SEM image of the purified fusion crystals. B) in vivo efficacy of activated Cry3Aa‐MIIA‐LL37‐P17 crystals was investigated by orally administration of *H. pylori*‐infected mice with activated crystals. The targeted delivery and release of LL‐37 at *H. pylori* infection site effectively reduced the *H. pylori* burden in the mouse stomach. Significantly, the treatment of activated Cry3Aa‐MIIA(D45E)‐LL37‐P17 crystals restored the balance of gut microbiome in the infected mice.

Herein, we report the design and generation of an antimicrobial *H. pylori*‐responsive crystal that is stable in the acidic gastrointestinal (GI) environment, thus allowing for oral administration. This multi‐module particle is composed of the Cry3Aa protein which serves as the in vivo crystal forming structural backbone, a metal/pH activated system for *H. pylori*‐triggered AMP release, the AMP, and a *H. pylori*‐targeting peptide. We demonstrate that this fusion crystal can protect the AMP from degradation, and specifically release it at the target site due to the pH elevation induced by *H. pylori* urease, resulting in the efficacious *H. pylori* eradication both in vitro and in vivo. Notably, treatment with the Cry3Aa‐AMP fusion crystals is found to successfully restore the gut microbiome altered by *H. pylori* infection (**Scheme** **1**).

## 2 Results and Discussion

### 2.1 Identification of LL‐37 as a Potent Anti‐*H. Pylori* Antimicrobial Agent

**Figure 1:**
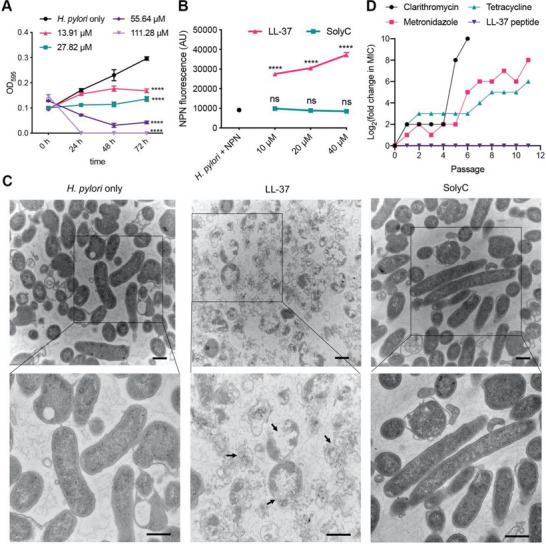
Anti‐*H. pylori* activity of LL‐37. A) Effect of LL‐37 on the growth of *H. pylori* SS1 at different concentrations and time points. The MIC of LL‐37 for *H. pylori* SS1 was ≈13.91 µm which is in agreement with previously published results.^[^ ^13c^ ^]^ The minimum bactericidal concentration was ≈111.28 µm. B) NPN uptake of *H*. *pylori* (10^8^ CFU) treated with different concentrations of LL‐37 or SolyC for 6 h. The increase in NPN fluorescence intensity indicates membrane permeation. Untreated bacteria incubated with NPN were used as negative control. Data are represented as mean ± SEM (*N* = 3). *P < 0.05, **P < 0.01, ***P < 0.001, ****P < 0.0001. ns, not significant. C) Transmission electron micrographs of *H. pylori* treated with 20 µm LL‐37 or SolyC for 24 h. The black arrows indicate rupture of the bacterial membrane. Scale bars: 500 nm. D) Development of antimicrobial resistance by *H. pylori* against clarithromycin, metronidazole, tetracycline, and LL‐37. Values are fold changes (in log_2_) of MIC compared to initial MIC at passage 0.

LL‐37 was found to be the most potent AMP against *H. pylori* SS1 (minimum inhibitory concentration (MIC) ≈13.91 µm among five AMPs that were previously reported to have anti‐*H. pylori* activity^[^ ^13^ ^]^ (**Figure** **1**A; Figure S1, Supporting Information). The anti‐*H pylori* activity of LL‐37 was further confirmed via an NPN (1‐*n*‐phenylnapthylamine) uptake assay^[^ ^22^ ^]^ and transmission electron microscopy (TEM). When *H. pylori* were exposed to different concentrations of LL‐37, a significant and dose‐dependent increase in fluorescence intensity was observed (Figure 1B), indicative of membrane permeation – a known mechanism of action for LL‐37. SolyC was used as a control and exhibited no such permeating ability at the concentrations tested. TEM micrographs of LL‐37‐treated *H. pylori* revealed severe bacterial membrane damage with numerous bacterial cells showing pores in their membranes and many lysed cells with depleted cell contents (Figure 1C). Given that LL‐37 has been reported to be cytotoxic at high concentrations,^[^ ^23^ ^]^ the cytotoxicity of LL‐37 on normal human gastric epithelial GES‐1 cells was thus evaluated. The IC_50_ of LL‐37 for GES‐1 cells was found to be ≈23.45 µm at 48 h and ≈24.76 µm at 72 h, which were twofold higher than the MIC (Figure S2, Supporting Information).

Since antibiotic resistance is the major cause of treatment failure, the susceptibility of LL‐37 to the development of antimicrobial resistance by *H. pylori* was investigated and compared to standard first‐line antibiotics (clarithromycin, metronidazole, and tetracycline). The evolution of resistance was rapid for all three antibiotics tested as indicated by the drastic fold increase in their MICs: 1024‐fold for clarithromycin, 256‐fold for metronidazole, and 64‐fold for tetracycline (Figure 1D). In contrast, *H. pylori* remained susceptible to LL‐37 throughout the 11 generations, lending credence to the suitability of LL‐37 as a potentially more effective therapeutic agent for the treatment of *H. pylori* infections.

### 2.2 Identification of a Selective *H. Pylori* Binding Peptide and Metal Ion‐Inducible Autocleavage (MIIA) Linker for Facile Cargo Release

**Figure 2:**
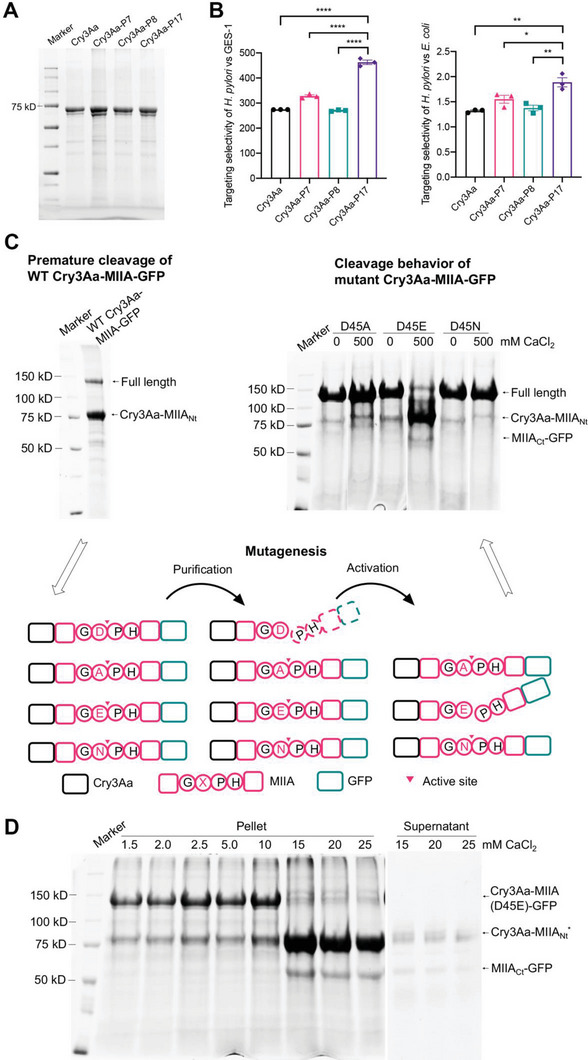
Selective binding of peptides toward *H. pylori* and cleavage behavior of the MIIA domain for cargo release. A) SDS‐PAGE of purified Cry3Aa crystals bearing a genetically fused 7‐mer peptide (Cry3Aa‐PX where X = 7, 8, or 17). B) The selectivity of the Cry3Aa‐PX (X = 7, 8, or 17) was determined by incubation of the Alexa‐647‐labeled crystals with *H. pylori*, *E. coli* or GES‐1 followed by flow cytometric analysis and calculated based on the percent of crystal‐bound *H. pylori* relative to that of GES‐1 (left) or *E. coli* (right). The selectivity for *H. pylori* relative to GES‐1 cells was adjusted for the surface area difference. Data are represented as mean ± SEM (*N* = 3). *P < 0.05, **P < 0.01, ****P < 0.0001. C) SDS‐PAGE gel revealed premature cleavage of wild type Cry3Aa‐MIIA‐GFP as indicated by the much diminished full length band at ≈150 kDa. The known cleavage site GDPH was mutated as illustrated in the schematic to produce Cry3Aa‐MIIA(D45A/E/N)‐GFP crystals. The cleavage behavior of the different Cry3Aa‐MIIA(D45A/E/N)‐GFP mutants under 0 or 500 mm CaCl_2_ was analyzed by SDS‐PAGE. D) SDS‐PAGE gel showcasing the cleavage efficiency of Cry3Aa‐MIIA(D45E)‐GFP crystals incubated in 1.5 – 25 mm CaCl_2_ for 16 h.

We next proceeded to identify an approach to specifically deliver LL‐37 to *H. pylori* to minimize indiscriminate membrane lysis that has been cited as one of the major roadblocks in the clinical translation of many AMPs.^[^ ^24^ ^]^ A previous study by Srinivasan et al. had identified three 7‐mer peptides (denoted as P7, P8, and P17) from phage‐displayed peptide library screens targeting cell surface epitopes of *H. pylori*, which exhibited high affinity and specificity toward different *H. pylori* isolates.^[^ ^25^ ^]^ Accordingly, we incorporated these binding sequences to the C‐terminus of Cry3Aa protein crystals as a genetic fusion (**Figure** **2**A) and evaluated their binding selectivity. Flow cytometric analyses indicated that the Cry3Aa crystals bearing the P17 peptide (Cry3Aa‐P17) showed the highest selectivity for *H. pylori* relative to GES‐1 cells as well as *E. coli* – another gram‐negative bacterium (Figure 2B).

Since LL‐37 needs to be folded into an active conformation to exert its antibacterial activity,^[^ ^23a^ ^]^ the impact of P17 conjugation on LL‐37′s bactericidal action was investigated using synthesized LL37‐P17 peptide. The LL37‐P17 peptide showed a similar MIC value (≈13.91 µm) as LL‐37′s for *H. pylori*, and was comparably effective in killing *H. pylori* (Figure S3, Supporting Information), indicating that the P17 conjugation did not adversely affect the antimicrobial activity of LL‐37.

Having settled on the AMP and targeting peptide, we then worked toward identifying a facile release system that could not only enable controlled release of LL‐37, but also be suitable for translational biosynthesis as a component of the antimicrobial Cry3Aa fusion crystals. We therefore sought a genetically‐encodable cleavage domain that could be activated by a common biologically compatible metal, such as calcium or magnesium to delink LL‐37 from Cry3Aa crystals. The MIIA cleavage domain isolated from *Vibrio coralliilyticu* appeared to satisfy these criteria. MIIA has been shown to undergo rapid autocleavage over a wide pH range in the presence of calcium or manganese ions at 2.5 mm concentration,^[^ ^26^ ^]^ which is close to the physiological concentration (2 mm) of calcium in blood plasma.^[^ ^27^ ^]^ We thus hypothesized that the MIIA domain should be well suited for promoting the facile disassociation of LL‐37 from Cry3Aa crystals in the gut.

To investigate the cleavage efficiency of the MIIA domain when fused to the Cry3Aa crystals, a construct comprising Cry3Aa, MIIA domain and the model cargo green fluorescent protein (GFP) was generated for the production of Cry3Aa‐MIIA‐GFP fusion crystals. Unexpectedly, this fusion crystal was found to undergo premature cleavage during production inside *Bt* cells and subsequent purification, resulting in the extensive loss of cargo protein (Figure 2C). Structural and biochemical studies of NopE1, a homolog of MIIA, have shown that the aspartyl‐proline bond of the Gly‐Asp‐Pro‐His (GDPH) motif was the cleavage site, and that the substitution of the aspartic acid (D) with asparagine (N) or alanine (A) abolished NopE1 cleavage, while the replacement with glutamic acid (E) had no impact.^[^ ^28^ ^]^ Hence, we generated Cry3Aa‐MIIA‐GFP crystals of the three GDPH motif mutants where Asp45 was replaced with alanine (D45A), glutamate (D45E) or asparagine (D45N), and explored the impact of these mutations on the crystal's cleavage activity (Figure 2C). As an initial investigation to ascertain the cleavability of the mutant MIIAs, Cry3Aa‐MIIA(D45X)‐GFP fusion crystals (X = A, E, N) were incubated with or without 500 mm CaCl_2_ for 16 h. SDS‐PAGE analysis indicated that the mutations of the Asp45 to Ala or Asn rendered the MIIA domain resistant to cleavage, even at high concentration of CaCl_2_. On the other hand, mutation to Glu yielded a mutant MIIA(D45E) with the desired controlled cleavage properties as evidenced by the dominant full‐length band of Cry3Aa‐MIIA(D45E)‐GFP crystals in the absence of CaCl_2_ and the truncated fragment bands at 500 mm CaCl_2_ (Figure 2C). We therefore employed this MIIA(D45E) mutant for our subsequent studies.

The Ca^2+^ ion‐inducible cleavage efficiency of the Cry3Aa‐MIIA(D45E)‐GFP was then evaluated at different concentrations of CaCl_2_, ranging from 1.5 – 25 mm, in order to determine the minimal Ca^2+^ concentration needed to achieve complete cleavage. Monitoring the cleavage by SDS‐PAGE showed that incubation with 15 mm CaCl_2_ for 16 h resulted in >99% cleavage (Figure 2D). Given the abundance of magnesium ions in cells, the cleavability of Cry3Aa‐MIIA(D45E)‐GFP crystals in 15 mm MgCl_2_ was also tested. Previous studies had indicated that magnesium ions could not induce MIIA cleavage,^[^ ^26b^ ^]^ and this was also true for Cry3Aa‐MIIA(D45E)‐GFP crystals (Figure S4, Supporting Information).

An intriguing observation from the aforementioned Ca^2+^ ion induced cleavage studies was that at 15 mm and higher CaCl_2_ concentrations, it appeared that the cleaved MIIA(D45E) C‐terminal‐GFP fragment (MIIA_Ct_‐GFP) remain associated with the Cry3Aa‐MIIA(D45E) N‐terminal fragment (Cry3Aa‐MIIA_Nt_*) as most of the MIIA_Ct_‐GFP was found in the cleaved pellet, while minimal cleavage products were observed in the supernatant (Figure 2D).

### 2.3 *H. Pylori*‐Responsive Release of the Cargo GFP Protein

Since the cargo protein needs to be released in order to act on its target, we wondered whether the release of the cleaved products from the crystals could be triggered by factors uniquely associated with *H. pylori*, such as the microenvironment of the bacterium. To this end, the release of MIIA_Ct_‐GFP from the Cry3Aa‐MIIA_Nt_*/MIIA_Ct_‐GFP complexes under relevant conditions was investigated.

**Figure 3:**
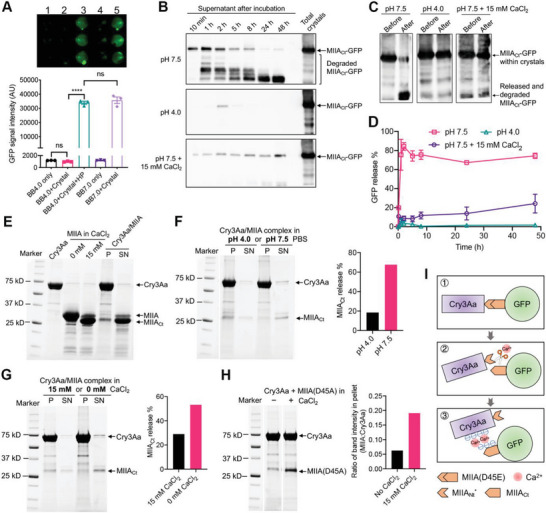
Release of GFP from the cleaved Cry3Aa‐MIIA(D45E)‐GFP crystals. A) Quantitation of GFP signal intensities of MIIA_Ct_‐GFP released into BB under different scenarios. Fluorescence of the supernatants of each experimental sample from the GFP release assay (upper) and their corresponding GFP signal intensities as measured using a TECAN (lower). Data are represented as mean ± SEM (*N* = 3). ****P < 0.0001. ns, not significant. B) Release of MIIA_Ct_‐GFP from cleaved crystals under different conditions was detected by western blot using anti‐GFP antibody. C) Crystal suspensions before and after incubation for 48 h in different buffers. Significant degradation of the released MIIA_Ct_‐GFP in the no Ca^2+^ pH 7.5 buffer was observed. D) Corresponding release profiles of the MIIA_Ct_‐GFP fragment in (B). Data are represented as mean ± SEM (*N* = 2). E) SDS‐PAGE analysis of the binding of wild‐type MIIA protein to Cry3Aa crystals in the presence of 15 mm CaCl_2_. Lanes 2–4 are protein controls. Lanes 5 and 6 are the Cry3Aa‐bound MIIA complex pellet (P) and supernatant (SN) after centrifugation. F,G) SDS‐PAGE analyses and corresponding bar plots on the release of MIIA_Ct_ from Cry3Aa‐bound MIIA complex in F) pH 4.0 or pH 7.5 PBS, and G) in 15 mm CaCl_2_ or no CaCl_2_, pH 7.5 buffer. P: pellet, SN: supernatant. H) Efficient binding of MIIA(D45A) protein to Cry3Aa crystals in the presence of CaCl_2_. I) Schematic illustration of the Ca^2+^‐medicated cleavage and binding of MIIA_Ct_‐GFP. Subfigures (E) and (F) are part of the same SDS‐PAGE gel sharing one molecular weight marker. The original gel image is provided in Figure S8 (Supporting Information). Subfigures (G) and (H) are derived from the same SDS‐PAGE gel and thus share one marker lane. The original gel image is provided in Figure S9 (Supporting Information). Subfigures (F) and (H) were produced by splicing the marker lane and the relevant lanes of the original gel image.

To mimic the *H. pylori*‐infected gastric environment, the Brucella Broth (BB) medium used for culturing *H. pylori* was acidified and inoculated with or without *H. pylori*. The Cry3Aa‐MIIA_Nt_*/MIIA_Ct_‐GFP complex generated by cleaving the Cry3Aa‐MIIA(D45E)‐GFP crystals with 15 mm CaCl_2_ was then added to the *H. pylori*‐free or *H. pylori*‐containing acidified BB. 24 h post‐incubation, the release of MIIA_Ct_‐GFP was determined by measuring the fluorescence intensity of the free MIIA_Ct_‐GFP protein in the BB solution. Interestingly, strong GFP signal was only observed in the acidified BB inoculated with *H. pylori*, indicating substantial release of the MIIA_Ct_‐GFP, whereas no MIIA_Ct_‐GFP was detected in the *H. pylori*‐free BB (**Figure** **3**A, lanes 1–3), thus suggesting that *H. pylori* is most likely the source of the stimulus that triggered the release of the MIIA_Ct_‐GFP protein from the fusion crystal.

It is known that *H. pylori* survives the acidic gastric environment by secreting urease to break down the gastric urea to ammonia, thereby neutralizing its surrounding acidic pH.^[^ ^29^ ^]^ This buffering mechanism also enables *H. pylori* to swim toward the surface of the gastric mucosa, which is at near neutral pH. We therefore surmised that the observed MIIA_Ct_‐GFP release might be due to the sudden elevation in the local pH of the BB medium brought upon by *H. pylori*. To verify the conjecture that the release was indeed pH‐dependent, the release of MIIA_Ct_‐GFP from the Cry3Aa‐MIIA_Nt_*/MIIA_Ct_‐GFP complex was examined in *H. pylori*‐free BB at pH 7.0. As expected, contrary to what was observed for *H. pylori*‐free BB at pH 4.0, intense green fluorescence comparable to that of the acidified BB inoculated with *H. pylori* was observed in *H. pylori*‐free BB pH 7.0 (Figure 3A, lanes 4–5). Although the exact mechanism is not clear, this distinct pH‐dependent cargo release from the crystal complexes suggested that the MIIA(D45E) domain could potentially serve as a *H. pylori*‐responsive trigger for the specific release of AMPs at *H. pylori* infection site.

### 2.4 Release Kinetics of the Cargo GFP Protein

While the release of the cleaved MIIA_Ct_‐GFP was observed at neutral pH post cleavage, we were confounded by the fact that such release was not detected during the process of Ca^2+^‐mediated cleavage in which the Cry3Aa‐MIIA(D45E)‐GFP crystals were incubated in 15 mm CaCl_2_ solution prepared in pH 7.5 buffer (100 mm Tris‐HCl) (Figure 2D). We thus hypothesized that CaCl_2_ might play a role in the binding and/or release of the cargo protein. To verify this hypothesis, Cry3Aa‐MIIA_Nt_*/MIIA_Ct_‐GFP complex was incubated in different buffers, namely, pH 4.0 buffer, pH 7.5 buffer, or pH 7.5 buffer supplemented with 15 mm CaCl_2_ for up to 48 h, and aliquots of the reaction samples were taken at different time points for the determination of release behavior and kinetics over time. While minimal release of MIIA_Ct_‐GFP was observed at pH 4.0, incubation in a neutral buffer led to a burst release of MIIA_Ct_‐GFP protein as much as 90% within 2 h (Figure 3B,D). It is worth mentioning that such a burst release of antimicrobials could be advantageous in fighting infections such as those of *H. pylori* since it enables a high initial concentration that is required to sufficiently kill the infectious organisms.^[^ ^30^ ^]^ Interestingly, the addition of 15 mm CaCl_2_ to the pH 7.5 buffer significantly slowed down the release of MIIA_Ct_‐GFP as only ≈20% was released even after 48 h (Figure 3B–D; Figure S5, Supporting Information), thus providing an explanation to why the MIIA_Ct_‐GFP fragment was not rapidly released from the cleaved fusion crystals during the Ca^2+^‐mediated cleavage process. Notably, the unreleased MIIA_Ct_‐GFP protein encapsulated inside the crystals was protected from degradation as indicated by the significant amount of its full‐length form remaining at the end of 48‐h incubation, whereas the released MIIA_Ct_‐GFP protein was gradually degraded after 10 min incubation (Figure 3B,C). This observation corroborated with previous studies demonstrating the distinct ability of Cry3Aa‐based crystal in conferring protection and stability to its cargo protein.^[^ ^21a^ ^]^

### 2.5 Interactions of the Cargo GFP Protein with Cry3Aa Fusion Crystals

In order to uncover the basis of the entrapment of the cleaved MIIA_Ct_‐GFP in the fusion crystals, we first focused on identifying the responsible interacting proteins. Previous report by Susan Ibe *et al.* had indicated that the cleaved MIIA C‐terminal (MIIA_Ct_) and N‐terminal (MIIA_Nt_) fragments might form a complex in the presence of high concentration of calcium ions (in their case is 25 mm) based on their experimental data.^[^ ^26a^ ^]^ We therefore reasoned that it might also be the case for the post‐cleavage association of MIIA_Ct_‐GFP with Cry3Aa‐MIIA_Nt_* crystals. To test this conjecture, we proceeded to first verify the interactions of free MIIA_Ct_ with its N‐terminal fragments in the presence of 15 mm CaCl_2._ Accordingly, wild‐type MIIA protein was purified and cleaved with CaCl_2_, and the cleaved products were analyzed by denaturing and native polyacrylamide gel electrophoresis. Disappointingly, a lower molecular weight band representing the MIIA_Ct_ fragment in the SDS‐PAGE gel was also observed in the native PAGE gel (Figure S6, Supporting Information), indicating that MIIA_Nt_ and MIIA_Ct_ did not form a complex – at least not at 15 mm CaCl_2_ concentration.

We thus moved on to the next most likely possibility, which was that MIIA_Ct_‐GFP might directly bind to Cry3Aa, based on the fact that previous studies from our laboratory have shown that Cry3Aa crystals contained wide negatively charged channels thus allowing for the entrapment of cationic AMPs and proteins, such as lysozyme and lipase A.^[^ ^20^, ^21^ ^]^ Given our previous observations that GFP did not bind to or get entrapped in Cry3Aa crystals, we speculated that it should be the MIIA_Ct_ motif that bound to the cleaved Cry3Aa‐MIIA_Nt_* crystals, or more specifically the Cry3Aa protein. To this end, purified wild‐type MIIA protein (Figure S7, Supporting Information) was incubated overnight with Cry3Aa crystals in 15 mm CaCl_2_, pH 7.5 buffer. To our delight, the MIIA_Ct_ fragment indeed bound to the Cry3Aa crystals (Figure 3E, lane 5). Moreover, the MIIA_Ct_/Cry3Aa complex exhibited similar pH‐dependent and Ca^2+^‐dependent properties in the release of the MIIA_Ct_ fragment (Figure 3F,G) that were observed for the MIIA_Ct_‐GFP release from the Cry3Aa‐MIIA_Nt_*/MIIA_Ct_‐GFP complex described above.

Having identified the binding partners, we sought to elucidate the binding mechanism behind this interaction. Since MIIA is an anionic protein (pI ≈3.96), it seemed counterintuitive that it might interact with the negatively charged channels of Cry3Aa crystals in a neutral environment. Nevertheless, given the aforementioned finding that calcium ions are critical to the Cry3Aa and MIIA_Ct_ binding, and together with the insights gleaned from previous studies that Ca^2+^ can facilitate the intermolecular cross‐linking of adjacent anionic molecules by forming protein‐Ca^2+^‐protein complexes,^[^ ^31^ ^]^ we hypothesized that the calcium ions could have facilitated the binding of MIIA protein to Cry3Aa via a Cry3Aa‐Ca^2+^‐MIIA interaction mediated by surface carboxylate and carbonyl residues. Under such a scenario, incubation of Cry3Aa crystals and MIIA protein in a buffer without any calcium ions would have abolished their binding. Indeed, when Cry3Aa crystals were incubated in calcium‐free pH 7.5 buffer with MIIA(D45A) protein, a non‐cleavable MIIA mutant to eliminate any potential effects of MIIA cleavage on the binding (Figure S7, Supporting Information), significantly less MIIA(D45A) proteins were found to bind to Cry3Aa crystals compared with that when in the presence of CaCl_2_ (Figure 3H). Further validation came from the significantly increased amount of MIIA(D45A) proteins bound to a more negatively charged Cry3Aa crystal (3A‐polyE), which was generated by replacing 11 channel residues with aspartate or glutamate,^[^ ^21e^ ^]^ in the presence of CaCl_2_ compared to that of Cry3Aa (Figure S10, Supporting Information).

Collectively, these data suggested that CaCl_2_ at 15 mm concentration could efficiently cleave the MIIA(D45A) domain within the fusion protein crystals while keeping the MIIA_Ct_‐cargo fragments bound to the Cry3Aa protein, thus entrapping the cargo inside the crystal (Figure 2D and Figure 3I) until it encounters an environment with low Ca^2+^ concentration and neutral pH (Figure 3A), such as the elevated pH catalyzed by the presence of *H. pylori* in the stomach. Owing to these distinct properties, and the fact that the 15 mm calcium concentration required to induce efficient cleavage of MIIA(D45E) in the fusion protein crystal is much higher than the 2 mm physiological concentration, we decided to use activated Cry3Aa‐MIIA(D45E)‐LL37‐P17 crystals (pre‐cleaved in 15 mm CaCl_2_) for the subsequent anti‐*H. pylori* experiments.

### 2.6 *H. Pylori‐*Responsive Release of LL‐37 from Activated Fusion Crystals and its Anti‐*H. Pylori* Activity

**Figure 4:**
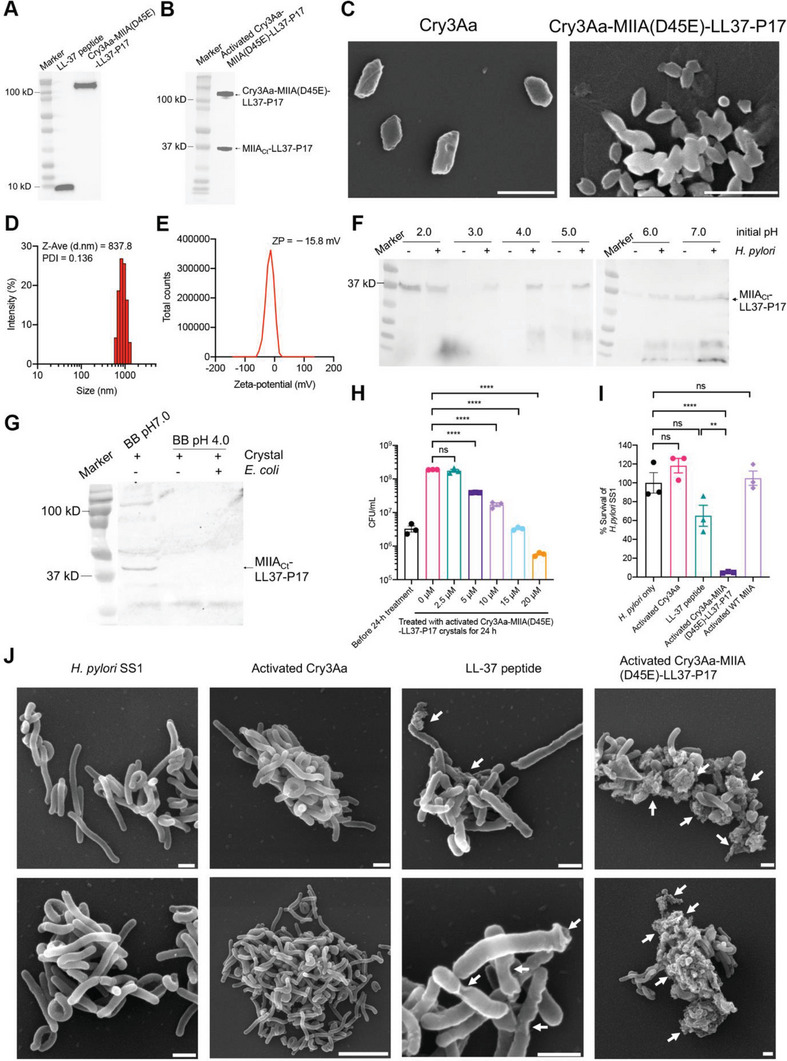
Characterization and antimicrobial activity of Cry3Aa‐MIIA(D45E)‐LL37‐P17 crystals. Western blot analyses of the presence of LL‐37 in purified Cry3Aa‐MIIA(D45E)‐LL37‐P17 crystals A) before activation and B) after activation with 15 mm Ca^2+^. C) SEM micrographs of native Cry3Aa and Cry3Aa‐MIIA(D45E)‐LL37‐P17 protein crystals. Scale bars: 2 µm. D) Size distribution and E) zeta‐potential of full‐length Cry3Aa‐MIIA(D45E)‐LL37‐P17 crystals. F,G) Western blot analysis on the release of LL‐37 from activated Cry3Aa‐MIIA(D45E)‐LL37‐P17 under different pH conditions in the presence of F) urease‐producing *H. pylori* or G) urease‐negative *E.coli*. H) Effect of activated Cry3Aa‐MIIA(D45E)‐LL37‐P17 crystals in the inhibition of *H. pylori* growth at different concentrations after 24 h incubation. I) Comparison of the anti‐*H. pylori* activity of Cry3Aa‐MIIA(D45E)‐LL37‐P17 crystals with LL‐37 at 10 µm after 24‐h incubation. The concentration of activated Cry3Aa‐MIIA(D45E)‐LL37‐P17 used was set to be equivalent to 10 μm LL‐37 peptide. Parallel treatment groups: activated Cry3Aa, activated wild‐type MIIA protein, and synthesized LL‐37 peptide were included for comparison. The survival of *H. pylori* in each treatment group was normalized against the untreated *H. pylori* control. Data are represented as mean ± SEM (*N* = 3). **P < 0.01, ****P < 0.0001. ns, not significant. J) Scanning electron micrographs of *H. pylori* after 24‐h treatment with activated Cry3Aa‐MIIA(D45E)‐LL37‐P17 or Cry3Aa crystal, or LL‐37 peptide. Arrows indicate damage to bacterial membrane. Scale bars: 5 µm. Note: Subfigure (G) is generated by splicing the marker lane of the colorimetric image and the chemiluminescent image of the same blot. The original images are provided in Figure S14, Supporting Information.

The desired construct comprising the genes encoding Cry3Aa, MIIA(D45E), LL‐37, and P17 targeting peptide was generated and the corresponding Cry3Aa‐MIIA(D45E)‐LL37‐P17 crystals were produced in *Bt*. The presence of LL‐37 in the purified full‐length fusion crystals as well as the Ca^2+^‐activated crystals was confirmed and quantitated by western blotting and densitometry (**Figure** **4**A,B; Figure S11, Supporting Information). The morphology of the purified fusion crystals was examined by scanning electron microscopy (SEM), which revealed that the crystals were uniform in size with mean length and width of ≈1.0 µm by 0.5 µm, similar to those of native Cry3Aa crystals (Figure 4C).^[^ ^20^ ^]^ Dynamic light scattering (DLS) further confirmed the uniformity and size of the full‐length fusion crystals (mean hydrodynamic diameter ≈837.8 nm, PDI = 0.136) (Figure 4D) and the activated crystals (mean hydrodynamic diameter ≈954.1 nm, PDI = 0.044) (Figure S12A, Supporting Information). Both the full‐length and activated fusion crystals showed similar zeta‐potentials, −15.8 mV and −15.2 mV, respectively (Figure 4E; Figure S12B, Supporting Information).

Next, the cleavage efficiency of Cry3Aa‐MIIA(D45E)‐LL37‐P17 crystals was evaluated. Similar to that of the Cry3Aa‐MIIA(D45E)‐GFP, cleavage could be achieved at 15 mm CaCl_2_ concentration (Figure 4B; Figure S13, Supporting information). We then investigated whether the release properties of the Cry3Aa‐MIIA(D45E)‐GFP crystal could be extended to this therapeutic construct. Toward this end, the release of MIIA_Ct_‐LL37‐P17 was evaluated for a wider range of pHs since the gastric pH of human varies from 1.5 to 6.6.^[^ ^32^ ^]^ At acidic pHs ranging from 3.0 – 6.0, the release of MIIA_Ct_‐LL37‐P17 occurred only in the presence of *H. pylori*, with the exception of the non‐specific release at pH 2.0, presumably due to the dissolution of the activated crystals. It has previously been reported that Cry crystals could be solubilized at pH ≤ 2.0.^[^ ^33^ ^]^ We then further verified that the release under acidic pH conditions was *H. pylori* specific by incubating the activated crystals with a urease‐negative bacterium – *E. coli*. As expected, the release of MIIA_Ct_‐LL37‐P17 at pH 4.0 occurred only in the presence of *H. pylori* (Figure 4F), but not *E. coli* (Figure 4G), thus reaffirming the specificity of this release. Based on these results, this construct appeared to be ideally suited for the delivery of LL‐37 for treating *H. pylori* infections. We rationalized that the acidic environment of the stomach would keep the MIIA_Ct_‐LL37‐P17 peptide in the crystals, and only release it upon encountering *H. pylori* − promoted by the pH elevation to neutral mediated by the action of *H. pylori* urease.

Consequently, the antimicrobial activity of the activated fusion crystals was assessed by incubating them with *H. pylori* SS1 for 24 h. The colony‐forming assay showed the fusion crystals inhibited the growth of *H. pylori* in a dose dependent manner (Figure 4H), and were significantly more effective in killing *H. pylori* compared with free LL‐37 peptides (Figure 4I; Figure S15, Supporting Information). Furthermore, it was found that the conjugation of P17 aided in enhancing the anti‐*H. pylori* activity of the activated Cry3Aa‐MIIA(D45E)‐LL37‐P17 crystals (Figure S16, Supporting Information). SEM was then performed to visualize the morphological changes on the bacterial membranes upon treatment with the activated Cry3Aa‐MIIA(D45E)‐LL37‐P17 crystals. Compared to the LL‐37 peptide treated sample, *H. pylori* treated with activated Cry3Aa‐MIIA(D45E)‐LL37‐P17 crystals appeared to incur more severe damage as debris of lysed bacterial cells and multiple blisters on the membrane surface were observed (Figure 4J). Such bacterial damage, however, was not found in the Cry3Aa crystal control sample, indicating that the observed membrane permeabilization is likely a result of the LL‐37 released from the activated fusion crystals.

### 2.7 Biological Fate and Toxicity of Orally Administered Cry3Aa‐MIIA(D45E)‐LL37‐P17 Crystals

**Figure 5:**
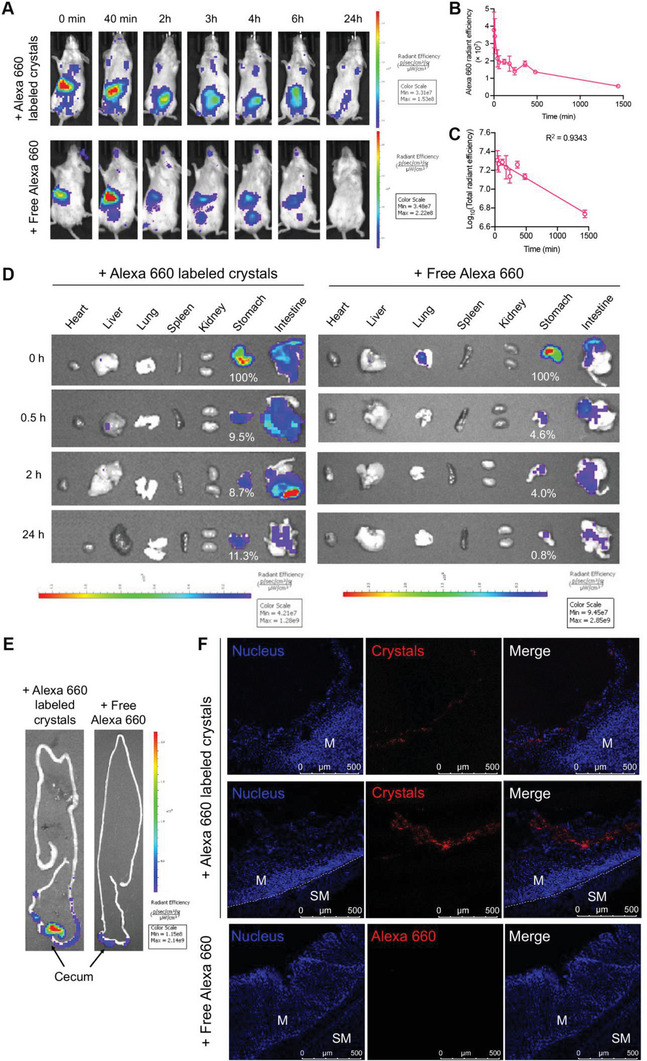
Retention and biodistribution of orally administered Cry3Aa‐MIIA(D45E)‐LL37‐P17 crystals. A) Representative fluorescence images of mice following the oral gavage of Alexa 660‐Cry3Aa‐MIIA(D45E)‐LL37‐P17 crystals or free Alexa 660 dye. B) in vivo clearance profile of the orally administered Alexa 660‐Cry3Aa‐MIIA(D45E)‐LL37‐P17 crystals, and C) the fitting of the corresponding clearance data (40 min to 24 h) to first‐order kinetics. Data are represented as mean ± SEM (*N* = 3). D) Ex vivo imaging of major organs at different time points: 0, 0.5, 2, and 24 h post‐administration. E) Accumulation of crystals or free Alexa 660 dye in cecum 2 h after oral administration. Confocal images showing F) the attachment of Alexa 660‐Cry3Aa‐MIIA(D45E)‐LL37‐P17 crystals to the luminal side of gastric mucosa. M: mucosa; SM: submucosa.

Having shown that Cry3Aa‐MIIA(D45E)‐LL37‐P17 crystals displayed the desired properties of an ideal antimicrobial agent, namely, targeting selectivity, stimuli‐responsive trigger, and facile cargo release in vitro, we proceeded to investigate the crystal's performance in vivo. Given that gastric retention is a major factor impacting the efficacy of oral therapeutics, the retention and biodistribution of Cry3Aa‐MIIA(D45E)‐LL37‐P17 crystals after oral administration were first evaluated. Mice were oral gavaged with Alexa 660‐labeled crystals or free Alexa 660 dye (control), and their in vivo fluorescence was tracked in real time for 24 h on a live imaging system. Intense fluorescence could be observed in the stomach of the Cry3Aa‐MIIA(D45E)‐LL37‐P17 crystal‐treated mice within 10 min of oral administration. This was followed by a rapid decrease of around 40% signal intensity in the first 40 min (**Figure** **5**A,B), presumably due to the quenching of the conjugated Alexa 660 dye in the gastric environment, and then a progressive reduction closely followed first‐order kinetics until the complete clearance of the crystals from the system after 24 h (Figure 5A–C). Ex vivo imaging of major organs of the fusion crystal‐treated mice sacrificed at different time points was also performed to elucidate the transit of these crystals through the gastrointestinal tract (GIT) and their subsequent biodistribution. Like most of the previously reported nanoparticles,^[^ ^34^ ^]^ the majority of the Cry3Aa‐MIIA(D45E)‐LL37‐P17 crystals exhibited a fast transit through the GI (Figure 5D) as shown by their accumulation in the cecum 2 h post‐ingestion and the much weakened fluorescence in the intestine at the 24‐h time point (Figure 5D,E). No apparent fluorescence signal was observed in any other organs. Meantime, it was noted that a considerable amount of orally administered crystals (9% – 11%) was retained in the stomach at the 24‐h time point (Figure 5D), which we hypothesized was due to the penetration of crystals into the gastric mucosa. This hypothesis was verified by analyzing the cryosections of the treated mouse stomach collected at the 2‐h period. Confocal images revealed the attachment of the orally administered Cry3Aa‐MIIA(D45E)‐LL37‐P17 crystals to the luminal side of the mucosa (Figure 5F; Figure S17, Supporting Information), thereby confirming the effective penetration and retention of crystals in the mucus layer where majority of the *H. pylori* bacteria reside.

Next, the biosafety of the activated fusion crystals was evaluated by oral gavaging C57BL/6 mice with 10, 30, and 60 mg kg^−1^ activated Cry3Aa‐MIIA(D45E)‐LL37‐P17 crystals once every other day for 18 doses. No obvious body weight change was observed during the treatment period (Figure S18A, Supporting Information). Furthermore, biochemical analyses of the serum levels of aspartate aminotransferase (AST), alanine aminotransferase (ALT), and creatinine of the blood collected 24 h after the last treatment revealed no significant difference between the crystal‐treated mice and the PBS control mice (Figure S18B–D, Supporting Information), indicating that the fusion crystals induced no toxicity to the mouse liver and kidneys. Furthermore, there was no meaningful change of organ index (organ weight/ body weight) for all collected organs, including heart, liver, spleen, lung, stomach, and kidneys (Figure S18E, Supporting Information) and no apparent organ damage based on examinations of the haematoxylin and eosin (H&E) stained organ tissues, suggesting that all organs preserved their architecture and there was no apparent organ damage (Figure S18F, Supporting Information). Periodic acid‐Schiff (PAS) staining of the fusion crystal‐treated mouse antrum and corpus tissues for all tested concentrations showed no difference in mucus thickness compared with that of the PBS control group, indicating that the Cry3Aa‐MIIA(D45E)‐LL37‐P17 crystals did not compromise the integrity of the mucosal barrier (Figure S18G,H, Supporting Information). These data indicate that the activated Cry3Aa‐MIIA(D45E)‐LL37‐P17 crystals are safe to mice even after repeated administrations.

### 2.8 In Vivo Anti‐*H. Pylori* Activity of Activated Cry3Aa‐MIIA(D45E)‐LL37‐P17 Crystals

**Figure 6:**
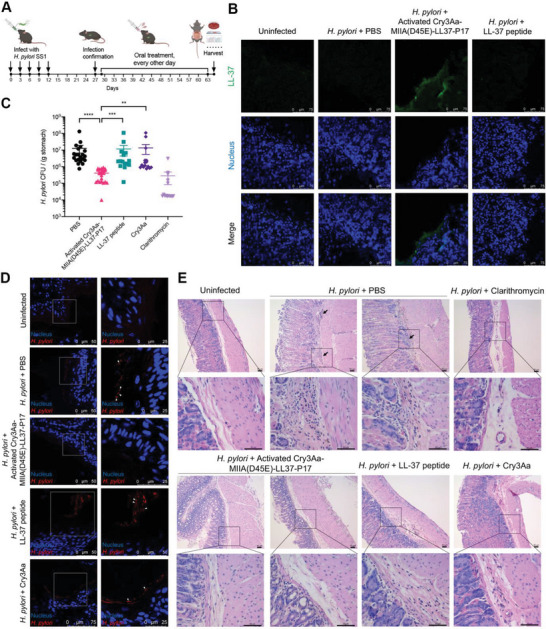
Anti‐*H. pylori* efficacy of activated Cry3Aa‐MIIA(D45E)‐LL37‐P17 fusion crystals in vivo. A) Treatment schedule for the efficacy studies of Cry3Aa‐MIIA‐LL37‐P17 to treat *H. pylori* infection. B) Immunofluorescence staining of LL‐37 in mouse stomach. Sections were stained with anti‐LL37 antibody (upper) and Hoechst 33 342 (middle). Merged images were shown (lower). Scale bars: 75 µm. C) *H. pylori* burden of infected mice treated with PBS (*N* = 21), activated Cry3Aa‐MIIA(D45E)‐LL‐37‐P17 (*N* = 22), LL‐37 peptide (*N* = 15), Cry3Aa (*N* = 15), and clarithromycin (*N* = 15). Data are represented as mean ± SEM. **P < 0.01, ***P < 0.001, ****P < 0.0001. D) Immunofluorescence staining of *H. pylori* in mouse stomach. Sections were stained with anti‐*H. pylori* antibody and Hoechst 33 342. Right panel corresponds to boxed region of left and white arrows indicate typical *H. pylori* cells. Scale bars are as shown. E) Histopathological analysis of *H. pylori*‐infected mice with different treatments. Arrows indicate typical immune cells infiltration. Scale bars: 50 µm.

C57BL/6 mice were oral gavaged with *H. pylori* SS1 and once the infection was confirmed (Figure S19, Supporting Information), *H. pylori*‐infected mice were orally administrated with PBS, clarithromycin (14.3 mg kg^−1^, 19 120 nmol kg^−1^),^[^ ^35^ ^]^ LL‐37 peptide (0.55 mg kg^−1^, 122.4 nmol kg^−1^), Cry3Aa crystals (21.93 mg kg^−1^, 300 nmol kg^−1^), or activated Cry3Aa‐MIIA(D45E)‐LL37‐P17 crystals (30 mg kg^−1^, 300 nmol kg^−1^, equivalent to 122.4 nmol kg^−1^ LL‐37), once every other day for a total of 18 doses (**Figure** **6**A). 48 h after the administration of the last dose, mice were sacrificed. To ascertain whether the activated Cry3Aa‐MIIA(D45E)‐LL37‐P17 crystals could successfully deliver LL‐37 to the stomach, we stained the differentially treated mouse gastric tissues with anti‐LL‐37 primary antibodies followed by Alexa 488‐labeled secondary antibodies and compared their fluorescence intensities. Stomach sections of mice treated with the activated fusion crystals showed the strongest LL‐37 signal, while the other treatment groups showed very weak or no fluorescence (Figure 6B), indicating the successful delivery of exogenous LL‐37 and reaffirming the activated fusion crystal's ability to overcome the mucosa barrier. Moreover, it is now known that, in addition to the predominant population of free‐swimming *H. pylori* in the surface mucus, a small population of *H. pylori* is found adhered to the epithelial surface or penetrates deep into gastric glands where they form microcolonies that contribute to chronic gastric infection.^[^ ^36^ ^]^ Although less widely distributed, fluorescence signals of LL‐37 could also be detected deeper in the gastric mucosa in the crystal‐treated stomach tissues (Figure S20, Supporting Information), demonstrating that the activated fusion crystal could deliver LL‐37 to the gland colonized *H. pylori*. It is also worth noting that even after 48 h, the orally administered LL‐37 could still be detected in the stomach barrier, demonstrating the robustness of the Cry3Aa delivery platform and the ability to protect its therapeutic cargo.

In keeping with the positive immunofluorescence staining of the exogenously delivered LL‐37 in the gastric mucosa, activated Cry3Aa‐MIIA(D45E)‐LL37‐P17 crystals were efficacious in the inhibition of *H. pylori* as demonstrated by the greatly reduced bacterial burden (≈30‐fold) in the stomach compared with LL‐37 peptide. Notably, this level of bacterial suppression was comparable to that of the antibiotic clarithromycin used as the positive control (Figure 6C). In addition, immunofluorescence staining of *H. pylori* in the crystal‐treated stomach tissues showed negligible signal, in contrast to those of the other treatment groups that displayed strong fluorescence, further corroborating the colony‐forming results (Figure 6D). Consistent with the aforementioned results, histopathological analysis of the H&E‐stained stomach tissues also showed no obvious inflammation for the activated Cry3Aa‐MIIA(D45E)‐LL37‐P17 crystals‐treated group (Figure 6E), and was similar to the stomach tissues of the uninfected mice, indicating that the activated Cry3Aa‐MIIA(D45E)‐LL37‐P17 crystals were able to resolve *H. pylori*‐induced inflammation. In addition, the mRNA expression of several cytokines analyzed by real‐time PCR revealed that the expression of IL‐1β and CXCL10 for the untreated group was significantly elevated by the end of the experimental period – week 8 post‐infection whereas treatment with the activated Cry3Aa‐MIIA(D45E)‐LL37‐P17 crystals appeared to aid in modulating this upregulation (Figure S21,Supporting Information).

Given the concern with antimicrobial resistance, we then asked ourselves whether the *H. pylori* extracted from the crystal‐ or antibiotic‐treated mouse tissues would still be susceptible to the same treatment antimicrobial. Toward this end, *H. pylori* recovered from the clarithromycin‐ and Cry3Aa‐MIIA(D45E)‐LL37‐P17‐ treated mice were cultured and tested for their susceptibility to their respective antimicrobials. Not surprisingly, the *H. pylori* recovered from clarithromycin‐treated mice showed a 4‐fold increase in MIC, indicative of resistance formation. In comparison, no resistance was observed in *H. pylori* recovered from the crystal‐treated mice as indicated by their susceptibility to LL‐37 peptide at the same MIC that inhibited the original *H. pylori* SS1 strain (Table S1, Supporting Information).

### 2.9 Effect of Activated Cry3Aa‐MIIA(D45E)‐LL37‐P17 Crystals on the Gut Microbiome

**Figure 7:**
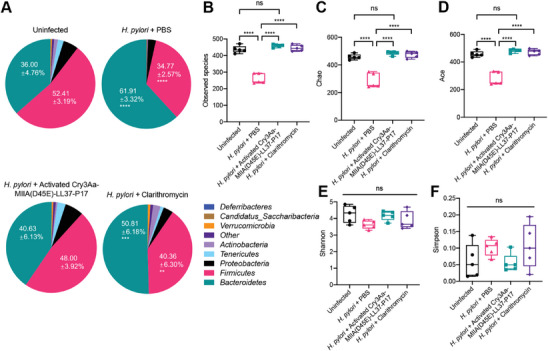
Effects of activated Cry3Aa‐MIIA(D45E)‐LL37‐P17 crystals on gut microbiome. A) Relative abundance of species on phylum level of the different experimental groups. Statistical tests were compared with uninfected group. B–F) Alpha diversity of different groups as measured by B) Observed species, C) Chao, D) Ace, E) Shannon, and F) Simpson indexes. Data are represented as mean ± SEM (*N* = 5). **P < 0.01, ***P < 0.001, ****P < 0.0001. ns, not significant.

It is now widely recognized that the gut microbiota is crucial to the optimal maintenance of host physiological processes, and that the alteration of gut microbial composition by factors such as antibiotics, and bacterial and viral infections negatively impacts human health.^[^ ^37^ ^]^ Hence, the effect of activated Cry3Aa‐MIIA(D45E)‐LL37‐P17 crystals as well as the other treatments on the gut microbiome of mice was evaluated by conducting 16S amplicon sequencing on the corresponding fecal DNA. As shown in **Figure** **7**A, gut microbiota dysbiosis was clearly observed in the untreated (*H. pylori* + PBS) and antibiotic‐treated *H. pylori*‐infected mice (*H. pylori* + clarithromycin), when compared with uninfected control mice. Untreated *H. pylori‐*infected mice exhibited significantly reduced gastric microbiota diversity and altered microbiota composition as evidenced by the disappearance of the *Verrucomicrobia* and *Tenericutes* phyla, and the shift in the *Firmicutes/Bacteroidetes* (F:B) abundance ratio 0.56:1 compared with 1.46:1 for uninfected control mice, respectively (Figure 7A; Table S2, Supporting Information). While treatment with clarithromycin could partially restore the gastric microbiota of the *H. pylori*‐infected mice, their gut microbial composition remained significantly altered, exhibiting substantial expansion in *Bacteroidetes* and concomitant contraction in *Firmicutes* – 50.81% and 40.36% respectively, compared with the levels of the uninfected mice at 36.00% and 52.41%. This change resulted in a shift from a dominance of *Firmicutes* to *Bacteroidetes*, and a reduction of the F:B ratio to 0.79:1 (Table S2, Supporting Information). In contrast, the gastric microbiota diversity as well as the community abundance on the phylum level of mice treated with the activated Cry3Aa‐MIIA(D45E)‐LL37‐P17 crystals showed no obvious differences compared with the levels in the uninfected mice (48.00 ± 3.92% for *Firmicutes*, 40.63 ± 6.13% for *Bacteroidetes* and F:B ratio 1.18:1), suggesting that the fusion crystals were able to reduce *H. pylori* colonization as well as reshape the *H. pylori*‐altered gut microbiome. This compares favorably to clarithromycin that though equally effective in killing *H. pylori* (Figure 6C) was unable to reverse the gut microbiota dysbiosis.

The impact of the treatment on the species diversity in each experimental group was also determined and compared by calculating the alpha diversity indices, including observed species, Chao, Ace, Shannon, and inverse Simpson indices. Untreated *H. pylori* infection negatively affected the species richness of the gut microbiome as indicated by the much lower alpha diversity indices, while the treatment with activated Cry3Aa‐MIIA(D45E)‐LL37‐P17 crystals or clarithromycin restored the gut microbiota's species richness (Figure 7B–F). Thus, the activated Cry3Aa‐MIIA(D45E)‐LL37‐P17 crystals not only inhibited the colonization of *H. pylori* in gastric mucosa, it also could reverse the altered gut microbiome caused by *H. pylori* infection.

LL‐37, the only cathelicidin in human plays a crucial role in defending the human body against foreign pathogens. Indeed, LL‐37 had been regarded as a therapeutic candidate in wound healing,^[^ ^38^ ^]^ herpes simplex virus 1 infection,^[^ ^39^ ^]^ and was evaluated in a Phase II clinical trial for treating venous leg ulcers.^[^ ^40^ ^]^ Most recently, Wang and colleagues showed that LL‐37 bound to SARS‐CoV‐2 S1 spike protein and the host receptor ACE2 with high affinity, effectively inhibiting S1 attachment to ACE2 thereby blocking SARS‐CoV‐2 from entering host cells.^[^ ^41^ ^]^ Nevertheless, there are few applications for treatment with LL‐37 by oral administration. There are reported delivery strategies rely on utilizing bioengineered bacteria carrying the gene encoding LL‐37 to express and secrete the AMP or its homolog, but not the direct delivery of the AMP itself.^[^ ^13^, ^42^ ^]^

The acidic and proteolytic environment of the stomach is a major barrier to any orally administered drugs, including peptides and proteins.^[^ ^43^ ^]^ Cry3Aa crystal is known for its stability in acidic pH,^[^ ^21a^ ^]^ and through the distinct Cry3Aa‐Ca^2+^‐MIIA interaction, LL‐37 could be entrapped inside the crystal at low pH and shielded from acidic and proteolytic degradation, thus enabling the delivery of effective concentration of the AMP to the mouse stomach.^[^ ^44^ ^]^ When combined with targeting specificity and *H. pylori*‐responsive release, these advantageous features collectively enable increased local concentration of the peptide at the infection site, resulting in enhanced anti‐*H. pylori* efficacy of the activated Cry3Aa‐MIIA(D45E)‐LL37‐P17 crystals (Figure 6B; Figure S20, Supporting Information). Moreover, the Cry3Aa crystal framework is highly amenable for modifications, as demonstrated by the successful biosynthesis of LL‐37 with Cry3Aa crystals. This enables the direct encapsulation of the AMP into the crystal without the need of a separate step, thus allowing for high cargo loading capacity, as well as making this approach extremely convenient and easy to implement.

While we have demonstrated that oral administration of activated Cry3Aa‐MIIA(D45E)‐LL37‐P17 crystals is able to restore the gut dysbiosis caused by *H. pylori* infection, the exact mechanism of action is unclear. Nevertheless, given that *H. pylori* is known to utilize different immune evasion strategies to enable its persistence in humans, including the downregulation of host release of antimicrobial peptides, which exert a major influence in shaping microbial communities in the gut,^[^ ^45^ ^]^ we believe it is the LL‐37 part that is the dominant contributor in promoting the restoration of the gut microbiome. Further insights can be drawn from numerous studies demonstrating that exogenous delivery of AMPs including cathelicidin could modulate dysbiosis in IBD mouse model,^[^ ^46^ ^]^ and more specifically, the studies by Sun and colleagues showing that defective production of CRAMP – the mouse ortholog of LL‐37 – exacerbates gluten‐induced small intestinal enteropathy (GIE) leading to increased intestinal permeability and GIE‐associated dysbiosis, but which could be corrected via exogenous CRAMP treatment.^[^ ^47^ ^]^

Given the promising results obtained for Cry3Aa‐MIIA(D45E)‐LL37‐P17 crystals for oral administration, the applications of the Cry3Aa fusion crystals in delivering LL‐37 or other therapeutic protein/peptide could be expanded to treat other gastrointestinal diseases in future investigations.

## 3 Conclusion

In this study, we describe the development of a modular therapeutic delivery system, the Cry3Aa‐MIIA(D45E)‐LL37‐P17 fusion crystal, for the oral delivery of the antimicrobial peptide LL‐37 to treat *H. pylori* infection. Each component of the fusion crystal possesses a distinct role that is optimized to provide the desired functionalities to achieve efficacious AMP delivery in an acidic environment. Said features include the crystal framework of Cry3Aa in conferring protection to LL‐37, the selective targeting of P17 peptide to *H. pylori*, and the controlled release of LL‐37 mediated by the distinct Ca^2+^‐ and pH‐dependent interactions of the MIIA and Cry3Aa proteins. We demonstrate that the activated Cry3Aa‐MIIA(D45E)‐LL37‐P17 crystals when orally administrated to *H. pylori*‐infected mice could effectively inhibit *H. pylori* growth as evidenced by the greatly reduced *H. pylori* burden and inflammation found in the mouse stomach tissues. Notably, treatment with these crystals could restore the dysbiotic gut microbiome in the *H. pylori*‐infected mice whereas clarithromycin, one of the standard antibiotics used to treat *H. pylori* infection, though equally effective in *H. pylori* inhibition, failed to do so. Collectively, our results suggest that LL‐37 can be considered as a potential therapeutic for the treatment of *H. pylori* infection and that the Cry3Aa platform can potentially aid in overcoming the barriers in oral drug delivery.

## 4 Experimental Section

### Animal Ethics Statement

C57BL/6 mice were housed in a temperature‐controlled room on a 10:14 h light/dark cycle maintained by the Laboratory Animal Service Centre, The Chinese University of Hong Kong (CUHK). All animal procedures were conducted with the approval of the Animal Experimentation Ethics Committee of CUHK (Ref No.: 16/018/MIS‐4‐B) and the Department of Health, the Government of the HKSAR under the Animals (Control of Experiments) Ordinance, Chapter 340 (20‐1085 in DH/HT&A/8/2/1 Pt.14).

### Bacterial Strain and Cell Culture

*H. pylori* standard strain Sydney strain 1 (SS1) was provided by the Department of Microbiology, Prince of Wales Hospital, CUHK. The bacteria were grown on horse blood agar plates made from Columbia blood agar base supplemented with 7% laked horse blood (ThermoFisher) and DENT selective supplements (Oxoid, UK), which contains a mixture of antibiotics, including vancomycin, trimethoprim, cefsulodin, and amphotericin B, in a microaerophilic environment for 5 d at 37 °C. For liquid culture, *H. pylori* colonies were inoculated into brucella broth (BB, BD BBL™) supplemented with 10% heat‐inactivated fetal bovine serum (FBS, HyClone) and DENT, in a microaerophilic environment for 20 h at 37 °C with 170 rpm shaking.

Human gastric epithelial cells (GES‐1) were maintained in Dulbecco's Modified Eagle medium (DMEM) supplemented with 10% FBS and 1x penicillin‐streptomycin (P/S) at 37 °C /5% CO_2_ in a humidified incubator.

### Antimicrobial Peptides

The antimicrobial peptides human cathelicidin (LL‐37), LL37‐P17, mouse cathelicidin (CRAMP), human α‐defensin 1 (HαD1), human β‐defensin 1 (HβD1), and the γ–core motif of tomato defensin (SolyC) were chemically synthesized by Pepmic Co. Ltd. (Suzhou, China) to a purity of >95% and supplied as lyophilized white powder. The HPLC‐purified peptides and their corresponding molecular weight were validated by mass spectrometry per the quality control reports provided by Pepmic. For the synthesized LL‐37, further verification of its correct molecular weight was performed using the Chinese University of Hong Kong's in‐house MALDI‐TOF mass spectrometry. Briefly, the lyophilized LL‐37 peptide was dissolved in 50% ACN / 0.1% TFA at 10 mg mL^−1^ and analyzed on a Bruker Ultraflextreme MALDI‐MS System (Figure S22, Supporting Information).

All the synthesized peptides were dissolved in deionized water to a concentration of 10 mg mL^−1^ and stored at −80 °C. The amino acid sequences of each peptide are listed in Table S3 (Supporting Information).

### Construction of Plasmids

For the construction of the plasmids used for the expression of different Cry3Aa crystal variants in *Bt*, the genes of interest were inserted into pHT315 vector. Briefly, for the production of the Cry3Aa‐P7, Cry3Aa‐P8, and Cry3Aa‐P17 fusion crystals, the nucleotide sequences encoding the three 7‐mer binding peptides (P7, P8, P17) (Table S4, Supporting Information) were incorporated into the reverse primers, and together with the forward primer were used to amplify the *cry3Aa* gene. The resultant gene inserts were then cloned into the *XhoI* and *KpnI* sites using Gibson Assembly (NEB). For the production of different Cry3Aa‐MIIA‐fusion crystals, the genes encoding the MIIA domain and LL‐37 were codon optimized for *Bt* expression and synthesized by Integrated DNA Technologies, USA, and the *gfp* gene was amplified from pEGFP‐N1 plasmid (Clontech). Gene cassettes containing genes encoding multiple proteins were produced by overlap PCR of the corresponding gene fragments and subcloned into the *BamHI* and *KpnI* sites downstream of the *cry3Aa* gene in the pHT315 vector. The plasmids for the production of Cry3Aa‐MIIA(D45A/E/N)‐GFP mutants were generated via site‐directed mutagenesis (Kapa Biosystem) using mutagenic primers containing the D45A/E/N mutations in MIIA.

To construct the plasmids for expression of wild type MIIA and MIIA(D45A) proteins in *E. coli*, the genes encoding wild type MIIA and MIIA(D45A) mutant were amplified and cloned into *BamHI* and *XhoI* sites of the pET28a vector using Gibson Assembly (NEB). The gene sequences of all constructs were verified by DNA sequencing (BGI).

### Expression and Purification of the Cry3Aa Fusion Protein Crystals

The aforementioned plasmids for *Bt* expression were transformed into *Bt 407‐OA* by electroporation, and the transformed cells were cultured in sporulation medium SSM to produce the corresponding crystals as previously described.^[^ ^21a^ ^]^ Cry3Aa fusion protein crystals were isolated by sucrose gradient centrifugation, and the purified protein crystals were washed extensively with ddH_2_O, except for the Cry3Aa fusion crystals containing the MIIA domain, which were washed with 500 mm EDTA pH 7.5 buffer. The purity of the purified Cry3Aa fusion protein crystals was examined using phase contrast microscope and analyzed by SDS‐PAGE.

The presence of LL‐37 in the full length and activated Cry3Aa‐MIIA(D45E)‐LL37‐P17 crystals was confirmed by western blotting using primary monoclonal anti‐LL‐37 antibody (mouse) (Santa Cruz sc‐166770, 1:250) and HRP conjugated rabbit anti‐mouse IgG secondary antibody (Abcam GR3383345, 1:5000).

To quantify the amount of LL‐37 in the purified Cry3Aa‐MIIA(D45E)‐LL37‐P17 crystals, serial dilutions of LL‐37 peptide for generating the standard curve and the fusion crystals were prepared and analyzed by western blotting using the same primary and secondary antibodies mentioned above.

### Expression and Purification of Soluble Proteins

pET28a plasmids harboring the genes encoding wild type MIIA and MIIA(D45A) mutant were transformed into *E. coli* BL21 (DE3) cells. The transformants were inoculated into Luria‐Bertani medium (LB) supplemented with kanamycin and grown at 37 °C until OD_600_ reached ≈0.6, at which time, overexpression of the corresponding proteins was induced using 0.2 mm IPTG at 25 °C, 220 rpm for 16 h. Pelleted cells were resuspended and lysed by sonication. Purified proteins were obtained using nickel affinity chromatography in 50 mm Tris‐HCl, 200 mm KCl, pH 8.0 buffer, and analyzed by SDS‐PAGE.^[^ ^26b^ ^]^ The purified proteins were dialyzed, concentrated, and stored at −80 °C for subsequent use.

### Antimicrobial Assays

*H. pylori* SS1 was cultured under standard culture conditions as described above. For the screening of different AMPs to identify the AMP with the most potent anti‐*H. pylori* activity, the synthesized peptides were twofold diluted in *H. pylori* culture medium (BB supplemented with 10% FBS) in a 96‐well plate. Cultured *H. pylori* at 10^8^ CFU per well were added to each well and incubated for 24, 48, and 72 h at 37 °C with shaking at 150 rpm. At each time point, *H. pylori* growth and its MIC were determined at OD_595_ using a microplate reader (Bio‐Rad).

For assaying the antimicrobial activity of Cry3Aa‐MIIA(D45E)‐LL37‐P17 crystals against *H. pylori*, purified crystals, including Cry3Aa‐MIIA(D45E)‐LL37‐P17, Cry3Aa‐MIIA(D45E)‐LL37, and Cry3Aa crystals, were first activated by incubating overnight at 37 °C in 100 mm Tris‐HCl pH 7.5 containing 15 mm CaCl_2_, followed by centrifugation. The pelleted calcium‐activated crystals were then tested for their antimicrobial activity. Briefly, cultured *H. pylori* were added into a 24‐well plate at 10^7^ CFU per well and then treated with 10 µm of either LL‐37, activated wild type MIIA protein, the aforementioned activated Cry3Aa crystals, or Cry3Aa‐MIIA(D45E)‐LL37‐P17 fusion crystals (equivalent to 10 µm LL‐37), and incubated at 37 °C with gentle shaking for 24 h. At the end of incubation, the treated cultures were serial diluted and spread onto *H. pylori‐*selective agar plates (Columbia blood agar base with DENT supplement) and incubated for 5 days to allow for colony formation. Colonies were manually counted and used to calculate CFU per mL of culture.

For the comparison of anti‐*H. pylori* activity of Cry3Aa‐MIIA(D45E)‐LL37 crystals with or without P17 conjugation, the antibacterial activity assay was performed similarly, except different concentrations of the activated Cry3Aa‐MIIA(D45E)‐LL37 or Cry3Aa‐MIIA(D45E)‐LL37‐P17 crystals were added to the cultured *H. pylori* seeded in a 24‐well plate.

As for the determination of MICs of *H. pylori* recovered from mice, single colonies from different mice were randomly picked from the agar plates used for in vivo colony‐forming assay, and cultured under standard condition (*N* = 4). 10^8^ CFU per well recovered *H. pylori* was pipetted into a 96‐well plate and then treated with serially diluted LL‐37 peptide or clarithromycin at 37 °C with shaking at 150 rpm for 24 h. *H. pylori* growth and MICs were determined at OD_595_ on a plate reader (Bio‐Rad).

### NPN Membrane Permeation Assay

The ability of LL‐37 or SolyC peptides to permeabilize the bacterial membrane was determined using 1‐*N*‐phenylnaphthylamine (NPN, Dieckmann) – a membrane potential‐sensitive fluorescent probe that exhibits increased fluorescence upon exposure to a hydrophobic environment.^[^ ^22^ ^]^ Briefly, a starter culture of *H. pylori* SS1 was grown for 16 – 18 h as described above. This cultured *H. pylori* (≈10^8^ CFU) was then incubated with different concentrations (10, 20, 40 µm) of either LL‐37 or SolyC peptides for 6 h. At the end of incubation, the treated bacteria were pelleted, fixed, washed, and then resuspended in a solution of 5 mm HEPES and 5 mm glucose pH 7.2 containing 22 g mL^−1^ of NPN. The samples were then transferred to a black 96‐well plate and the NPN fluorescence intensity was measured using an Infinite M1000 Pro (TECAN, λ_ex_ = 350 nm, λ_em_ = 420 nm).

### Cytotoxicity of LL‐37 on GES‐1 Cells

GES‐1 cells seeded at 3000 cells per well in 96‐well plates (Nunc) were incubated at 37 °C/ 5% CO_2_ with serial dilutions of LL‐37 (5 – 45 µm) for either 48 or 72 h. At the end of the incubation period, MTT (One Scientific, UK) reagent was added to each well at a final concentration of 0.5 mg mL^−1^ and incubated for another 4 h. The purple formazan was dissolved by DMSO and the absorbance at 565 nm was recorded on an Infinite M1000 Pro (TECAN) plate reader. The viability of GES‐1 was calculated using the following formula:

(1) $$\begin{array}{ccc} & & \%\;\mathrm{Viability}\;\mathrm{of}\;\mathrm{GES}-1=\left[{\mathrm{OD}}_{565\;\mathrm{nm}}\left(\text{treated-blank}\right)/\right.\\ & & \;\left.{\mathrm{OD}}_{565\;\mathrm{nm}}\left(\text{non-treated-blank}\right)\right]\times 100\% \end{array}$$

### Antimicrobial Resistance Development Assay

To determine the initial MICs of clarithromycin (TCI), metronidazole (Sigma), tetracycline (Scharlab) and LL‐37, *H. pylori* (≈10^8^ CFU) were incubated with different concentrations of the antimicrobial agents at 37 °C for 24 h with gentle shaking and their growth was determined by measurement of OD_595_ on a plate reader (Bio‐Rad) (designated as passage 0). Bacteria from the highest concentration below MICs were cultured and inoculated into a subsequent plate containing serial concentrations of antimicrobial agents for the next assessment. The process was repeated for up to 11 passages and the fold changes of the MIC were recorded.

### Transmission Electron Microscopy (TEM)

LL‐37 or SolyC peptides (20 µm) were incubated with *H. pylori* SS1 (10^8^ CFU) for 24 h. The treated bacterial cells were pelleted and washed in PBS twice, then fixed in 2.5% glutaraldehyde and embedded in low melting temperature agarose. The embedded bacteria were cut into 1‐mm cubes, post‐fixed in 2% OsO_4_ for 2 h, followed by dehydration in a graduated series of ethanol. The dehydrated cells were then embedded in pure spur, sectioned (≈70 nm per section) and post‐stained for 10 min in 2% aqueous uranyl acetate followed by 5 min in Reynold's lead citrate. The samples were then imaged using a H‐7650 TEM (Hitachi).

### Scanning Electron Microscopy (SEM)

To examine the size and morphology of Cry3Aa and Cry3Aa‐MIIA(D45E)‐LL37‐P17 crystals, 100 µg mL^−1^ purified crystals were gradually dehydrated in ethanol (25, 50, 75, 95%), and 1 µL of the preparation was then added to a glass cover slip and air dried overnight for imaging.

To examine the morphological changes of *H. pylori* SS1 treated with activated Cry3Aa‐MIIA(D45E)‐LL37‐P17 crystals, 10^9^ CFU *H. pylori* were seeded with either 15 µm LL‐37 peptide, 1 µm activated Cry3Aa‐MIIA(D45E)‐LL37‐P17 crystals, or 1 µm activated Cry3Aa (control) for 24 h at 37 °C with gentle shaking. At the end of incubation, *H. pylori* were pelleted and washed with PBS, then fixed with 2.5% glutaraldehyde for overnight at 4 °C. The bacteria were dehydrated stepwise in a series of increasing concentration of ethanol, followed by incubation with hexamethyldisilazane (HMDS), and air dried overnight. Prepared samples were sputter‐coated (Edwards S150B) with gold palladium prior to imaging on an FEI Prisma E Environmental scanning electron microscope (Thermo Fisher).

### Dynamic Light Scattering and Zeta Potential Measurement

The size and zeta potential of the crystals were determined by Malvern Nano ZS90 (Malvern instrument, UK) at 25 °C. Full‐length or activated Cry3Aa‐MIIA(D45E)‐LL37‐P17 protein crystals were resuspended in PBS at 150 µg mL^−1^ and 50 µg mL^−1^ for dynamic light scattering (DLS) and zeta potential measurement, respectively.

### Binding Specificity and Selectivity of Cry3Aa Crystals Bearing Targeting Peptides

Purified Cry3Aa and Cry3Aa‐PX (X = 7, 8, and 17) fusion crystals were labeled with Alexa Fluor™ 647 maleimide (ThermoFisher). *H. pylori* and *E. coli* were stained with Hoechst 33342. The labeled crystals (1 µm) was incubated with either Hoechst 33342‐stained bacteria seeded at 10^8^ CFU in a 12‐well plate, or GES‐1 cells seeded at 10^5^ cells per well in a 6‐well plate for 4 h at 37 °C. At the end of the incubation period, the treated GES‐1 cells were washed with 2 U mL^−1^ heparin in PBS, trypsinized, and washed extensively with PBS while the treated bacteria were fixed in 4% paraformaldehyde followed by extensive washing. Cells were then analyzed on a BD FACSVerse flow cytometer. The binding specificity of each crystal construct was determined based on the percentage of cells with bound labeled crystals.

The targeting selectivity, defined as the selectivity of Cry3Aa or Cry3Aa‐PX crystals for *H. pylori* over GES‐1 cells or *E. coli* was determined by flow cytometric analysis and calculated based on the percent of crystal‐bound *H. pylori* relative to that of the other organism.

For GES‐1 cells, the *H. pylori* targeting selectivity was adjusted for the difference in their surface areas. The formula used was:

(2) $$\begin{array}{ccc} & & H.\mathrm{pylori}\;\mathrm{targeting}\;\mathrm{selectivity}=\left(\%\;\mathrm{of}\;H.\;\mathrm{pylo}\mathrm{ri}\;\mathrm{binding}\;\mathrm{with}\;\mathrm{crystals}/\right.\\ & & \;\left.\%\;\mathrm{of}\;\mathrm{GES}-1\;\mathrm{binding}\;\mathrm{with}\;\mathrm{crystals}\right)\left(\mathrm{surface}\;\mathrm{area}\;\mathrm{of}\;\mathrm{GES}-1/\right.\\ & & \;\left.\mathrm{surface}\;\mathrm{area}\;\mathrm{of}\;H.\mathrm{pylori}\right) \end{array}$$

### Activation of MIIA‐Containing Cry3Aa Fusion Crystals

For the identification of the optimal construct with both metal‐induced cleavage activity and minimal auto‐release of the cargo protein from the Cry3Aa fusion crystals, Cry3Aa‐MIIA‐GFP mutants containing a mutation of Asp45 in the MIIA domain to either glutamate (D45E) or asparagine (D45N) or alanine (D45A) were incubated with 100 mm Tris‐HCl pH 7.5 buffer with or without 500 mm CaCl_2_ at 37 °C overnight with 1000 rpm shaking in a thermomixer (Eppendorf). At the end of incubation, the reaction mixtures were analyzed on SDS‐PAGE gel to determine the relative levels of cleaved and uncleaved products.

To optimize the concentration of CaCl_2_ for the cleavage of Cry3Aa‐MIIA(D45E)‐GFP, the fusion crystals were incubated with different concentrations of CaCl_2_ (1.5 – 25 mm) in 100 mm Tris‐HCl pH 7.5 buffer at 37 °C overnight with 1000 rpm shaking in a thermomixer (Eppendorf). The suspensions were then centrifuged, and the corresponding supernatants and pellets were subjected to SDS‐PAGE analysis.

The cleavage of Cry3Aa‐MIIA(D45E)‐GFP in 15 mm MgCl_2_ was performed similarly as described above, except that the CaCl_2_ in the cleavage solution was replaced with MgCl_2_.

The cleavage assay to determine the minimal CaCl_2_ concentration to induce cleavage of Cry3Aa‐MIIA(D45E)‐LL37‐P17 crystals followed the same procedure as that for Cry3Aa‐MIIA(D45E)‐GFP, except in this case, the resultant crystal pellets were analyzed by western blotting using primary monoclonal anti‐LL‐37 antibody.

The activation of Cry3Aa‐MIIA(D45E)‐LL37‐P17 was initiated by incubating the purified crystals in 100 mm Tris‐HCl, 15 mm CaCl_2_, pH 7.5 buffer at 37 °C overnight with 1000 rpm shaking in a thermomixer (Eppendorf).

### Release of Cargo Protein from Cry3Aa Fusion Crystals

To evaluate the *H. pylori*‐responsive release of GFP from calcium‐activated Cry3Aa‐MIIA(D45E)‐GFP crystals under different physiologically relevant conditions, the Cry3Aa‐MIIA(D45E)‐GFP fusion crystals were first activated by incubating the Cry3Aa‐MIIA(D45E)‐GFP fusion crystals overnight in 100 mm Tris‐HCl, 15 mm CaCl_2_, pH 7.5 buffer at 37 °C. The suspension was then centrifuged and the pellet (denoted as activated crystals hereafter) was resuspended in either BB pH 7.0 (regular BB) or acidified BB pH 4.0 (adjusted using HCl). The suspensions were then added to a 96‐well plate containing regular BB or acidified BB supplemented with 10 mm urea, inoculated with or without *H. pylori*. The reaction plate was incubated at 37 °C for 24 h with gentle shaking. At the end of incubation, samples were transferred to microcentrifuge tubes and centrifuged at 15 000 g for 10 min. The supernatant of each sample was then pipetted to a black 96‐well plate and the fluorescence determined using an Infinite M1000 Pro (TECAN, λ_ex_ = 488 nm and λ_em_ = 510 nm).

The release efficiency of GFP from Cry3Aa‐MIIA(D45E)‐GFP was determined by first activating 1 mg mL^−1^ Cry3Aa‐MIIA(D45E)‐GFP crystals overnight as described above. The activated crystals were resuspended in 500 µL of either 100 mm Citric acid/NaOH (pH 4.0) or 100 mm Tris‐HCl (pH 7.5) buffer with or without 15 mm CaCl_2_ at 37 °C for up to 48 h. 50 µL of suspension was collected from each reaction mixture at different time points (0, 1, 3, 5, 8, 24, and 48 h) and centrifuged to separate the supernatant and the pellet. Images of the corresponding GFP fluorescent pellets were captured using ChemiDoc (Bio‐Rad) while 20 µL of the corresponding supernatant was used for western blotting. The MIIA_Ct_‐GFP released into the supernatant was detected using anti‐GFP (rabbit) primary antibody (Rockland 600‐401‐215, 1:2000) and HRP conjugated secondary antibody of goat anti‐rabbit IgG (Bio‐Rad 170–6515, 1:5000). The band intensity (BI) was quantitated using Bio‐Rad ImageLab software, and the percentage of GFP release was calculated using the following formula:

(3) $$\begin{array}{ccc} & & \%\;\mathrm{GFP}\;\mathrm{release}=\mathrm{BI}\left[\mathrm{released}\;{\mathrm{MIIA}}_{\mathrm{Ct}}-\mathrm{GFP}\;\mathrm{in}\;\mathrm{the}\;\mathrm{supernatant}\right]/\\ & & \;\mathrm{BI}\;\left[\mathrm{total}\;{\mathrm{MIIA}}_{\mathrm{Ct}}-\mathrm{GFP}\;\mathrm{in}\;\mathrm{crystals}\right]\times 100\% \end{array}$$

To investigate whether the release of LL‐37 from Cry3Aa‐MIIA(D45E)‐LL37‐P17 crystals was due to the elevated pH in the local environment mediated by the *H. pylori*‐produced urease, CaCl_2_‐activated Cry3Aa‐MIIA(D45E)‐LL37‐P17 crystals (10 µm) were incubated in acidic (pH 2.0 – 6.0 adjusted using HCl) and neutral pH conditions as described above for Cry3Aa‐MIIA(D45E)‐GFP crystals in the presence of *H. pylori* or *E. coli*. At the end of the incubation, the amount of LL‐37 released was determined by western blotting using monoclonal primary anti‐LL‐37 (mouse) antibody (Santa Cruz sc‐166770, 1:250) and HRP conjugated secondary antibody of rabbit anti‐mouse IgG (Abcam GR3383345, 1:5000).

### Binding Analysis of MIIA to Cry3Aa Crystal

To determine whether MIIA C‐terminal and N‐terminal fragments formed a complex after cleavage, purified wild type MIIA protein was incubated at 25 °C in 100 mm Tris‐HCl, pH 7.5 buffer supplemented with either 0 or 15 mm CaCl_2_ for 90 min. The resultant cleavage products were then subjected to denaturing SDS‐PAGE and native PAGE analyses.

To evaluate the mechanism of MIIA‐cargo protein binding with Cry3Aa crystals after activation, Cry3Aa protein crystals were incubated with wild type MIIA protein at a 1:5 molar ratio in 100 mm Tris‐HCl, 15 mm CaCl_2_, pH 7.5 buffer overnight at 10 °C with gentle shaking in a thermomixer (Eppendorf). The protein‐crystal mixtures were pelleted and incubated in 20 µL pH 7.4 PBS, or pH 4.0 PBS (adjusted using HCl), or 100 mm Tris‐HCl pH 7.5 with or without 15 mm CaCl_2_ for 2 h.

To ascertain the role of calcium ions on the binding of MIIA protein to Cry3Aa crystals, the non‐cleavable MIIA (MIIA(D45A)) protein and Cry3Aa or more negatively charged 3A‐polyE crystals were incubated at a 1:5 molar ratio in 100 mm Tris‐HCl, pH 7.5 buffer with or without 15 mm CaCl_2_ at 10 °C overnight in a thermomixer (Eppendorf) with shaking at 600 rpm. At the end of incubation, the suspensions were centrifuged and their supernatants and pellets were subjected to SDS‐PAGE analysis. The band intensity was quantitated using Bio‐Rad ImageLab software, and the percentage of MIIA_Ct_ release was calculated as follows:

(4) $$\begin{array}{ccc} & & \%\;{\mathrm{MIIA}}_{\mathrm{Ct}}\;\mathrm{release}=\mathrm{BI}\left[\mathrm{released}\;{\mathrm{MIIA}}_{\mathrm{Ct}}\;\mathrm{in}\;\mathrm{the}\;\mathrm{supernatant}\right]/\\ & & \;\mathrm{BI}\left[\mathrm{total}\;{\mathrm{MIIA}}_{\mathrm{Ct}}\;\mathrm{in}\;\mathrm{the}\;\mathrm{supernatant}\;\mathrm{and}\;\mathrm{pellet}\right]\times 100\% \end{array}$$

### In Vivo and Ex Vivo Imaging

For in vivo imaging studies, Cry3Aa‐MIIA(D45E)‐LL37‐P17 crystals were labeled with Alexa Fluor™ 660 NHS Ester (Thermo Fisher). Briefly, 5 mg mL^−1^ fusion crystals were resuspended in 0.2 m sodium bicarbonate (pH 8.0) and incubated with Alexa Fluor™ 660 NHS Ester in a crystal: dye molar ratio of 1:2, followed by 1 h continuous mixing at room temperature. The reaction mixture was centrifuged at 15000*g* for 5 min, and the Alexa 660‐labeled fusion crystals were washed extensively with ddH_2_O for three times and stored at 4 °C prior to use. Adult female BALB/c mice were fasted overnight to minimize autofluorescence from the mouse chow before their oral administration with 30 mg kg^−1^ of Alexa 660‐labeled fusion crystals or free Alexa 660 dye (control).

To elucidate the fate of Cry3Aa‐MIIA(D45E)‐LL37‐P17 crystals in mice, whole body imaging using an IVIS Spectrum in vivo imaging system was performed to monitor the fluorescence signal of crystals over time (0, 10, 40 min, and 1, 2, 3, 4, 6, 8, 24 h post administration). The fluorescence signals were quantitated using Living Image software.

For the biodistribution of the fusion crystals, mice were sacrificed at different time points post administration (0, 0.5, 2, and 24 h) and their organs were isolated for ex vivo imaging using an IVIS Spectrum imaging system.

To ascertain whether the Cry3Aa‐MIIA(D45E)‐LL37‐P17 crystals were attached to gastric epithelium after oral administration, mice stomach harvested at 2 h were fixed and cryo‐sectioned. Hoechst 33342 (Life Technologies) was used to stain cell nucleus. Sections were evaluated by confocal microscopy (Leica SP8).

### In Vivo Toxicity

C57BL/6 mice (5‐week‐old male) were randomly assigned into groups and oral gavaged with PBS (*N* = 4) or different dosages (10, 30, and 60 mg kg^−1^) of activated Cry3Aa‐MIIA(D45E)‐LL37‐P17 crystals (*N* = 5) once every other day for 18 doses. Mice were sacrificed 1 day after receiving the last treatment and their organs were harvested. Blood was collected in heparin‐rinsed 1.5 mL microtube, and the levels of aspartate aminotransferase (AST), alanine aminotransferase (ALT), and creatinine in serum were determined using Stanbio AST/GOT, Stanbio ALT/GPT, and Stanbio Direct Creatinine LiquiColor kits, respectively. Major organs, including heart, liver, spleen, lung, kidneys, and stomach of each mouse were weighed and fixed for histological analysis. The organ index was calculated based on the following formula:

(5) Organindex=Organweight/Bodyweight

Paraffin sections of different tissues were stained with haematoxylin and eosin (H & E) for histological examination. For the measurement of mucus layer thickness, the paraffin‐embedded stomach tissues were stained with periodic acid‐Schiff (PAS) and counterstained with haematoxylin following manufacturer's instruction, and imaged by light microscopy (Leica DM2000). The thickness of the mucus layer was measured perpendicularly from the epithelial surface to the outermost part of the mucus secreting layer. For each mouse, more than ten measurements from slides of two different portions of stomach tissues were examined and the results were shown as the ratio of thickness of the mucus‐secreting layer to the thickness of total mucosa.

### In Vivo Efficacy Studies of AMP Fusion Protein Crystals toward H. Pylori Infection in Mouse Model

After overnight fasting, C57BL/6 mice (5‐week‐old male) were oral gavaged with either brain heart infusion (BHI) or *H. pylori* SS1 (10^9^ CFU per mouse) in BHI once every three days for a total of five doses. Two weeks post infection, one mouse gavaged with BHI and two mice infected with *H. pylori* SS1 were sacrificed and their stomachs were harvested to confirm *H. pylori* infection by PCR. Briefly, mouse stomach genomic DNA was extracted using a DNA purification kit (Promega, San Luis Obispo, CA) according to the manufacturer's instruction. Extracted DNA (200 ng) and primers specific for detecting *H. pylori* 16S rDNA (HP_F: 5′‐TTTGT TAGAG AAGAT AATGA CGGTA TCTAA C‐ 3′; HP_R: 5′‐CATAG GATTT CACAC CTGAC TGACT ATC‐3′) were used in the PCR reaction. PCR result was analyzed on a 2% agarose gel (Bio‐Rad).

After *H. pylori* infection was confirmed, mice were randomly assigned into groups (*N* ≥ 15) and oral gavaged with PBS (*N* = 21), clarithromycin (14.3 mg kg^−1^, *N* = 15),^[^ ^35^ ^]^ or 300 nmol kg^−1^ of either Cry3Aa (*N* = 15) or activated Cry3Aa‐MIIA(D45E)‐LL‐37‐P17 (with 122.4 nmol kg^−1^ LL‐37 peptide loaded, *N* = 22) crystals prepared as previously described, and 122.4 nmol kg^−1^ LL‐37 peptide (*N* = 15) once every other day for 18 doses.

Two days after the final treatment, 3–4 fresh fecal pellets were snap‐frozen in liquid nitrogen and stored at −80 °C until use. Mice were sacrificed and their stomach tissues were harvested, cut, and washed in PBS. Half of the fresh stomach tissues were used for colony‐forming assay while the remaining half was split into four portions. One portion was immersed in 30% sucrose/PBS overnight, embedded in O.C.T compound (SAKURA,) and stored at −80 °C for cryo‐sectioning, another portion was snap‐frozen in liquid nitrogen and stored at −80 °C for RNA extraction, while the remaining two portions were fixed and embedded in paraffin for histopathological examinations.

### Colony‐Forming Assay

For the quantification of *H. pylori* colonization in mouse stomach, stomach tissues were homogenized, then serially diluted and plated on *H. pylori‐*selective agar plates (Columbia blood agar base with DENT supplement) and incubated for 5 days to allow colony formation. Colonies were manually counted and the infection level was calculated as CFU per gram of stomach tissue. Urease, oxidase, and catalase tests, and gram staining were performed on selected colonies for the confirmation of *H. pylori's* identity.

### Histopathological Evaluation of Inflammation

Paraffin‐embedded sections of stomach were first deparaffinized in xylene and hydrated in graded ethanol. Sections were stained in Mayer's hematoxylin and 0.5% aqueous eosin, and then imaged by light microscopy (Leica DM2000).

### RNA Extraction and Real‐Time PCR

The total RNA was extracted from mouse stomach tissues using TRIzol reagent (Invitrogen). Reverse transcription was performed using iScript™ advanced cDNA synthesis kit (Bio‐Rad). Synthesized cDNAs were probed with specific primers listed in Table S5 (Supporting Information) using SsoAdvanced™ universal SYBR green supermix (Bio‐Rad) and real‐time PCR system (Bio‐Rad CFX96). The mRNA expression levels were normalized to GAPDH and quantitated using 2^−∆∆C^_T_ method.

### Immunofluorescence Staining

O.C.T compound embedded tissues were sectioned with a thickness 10 µm (Leica CM1950), washed in PBS, and post‐fixed in methanol. The samples were then blocked in 5% goat serum/3% BSA/0.1% Triton X‐100/PBS blocking solution, followed by incubation with primary anti‐LL37 (mouse, Santa Cruz sc‐166770) or anti‐*H. pylori* (rabbit, Abcam ab7788) antibodies at 4 °C for overnight. Alexa Fluor™ 488 anti‐mouse IgG (Abcam) or Alexa Fluor™ 647 anti‐rabbit IgG (Abcam) were used as secondary antibodies, respectively. Hoechst 33342 (Life Technologies) was used to stain cell nucleus. Sections were evaluated by confocal microscopy (Leica SP8).

### Analysis of Gut Microbiome

Two days after the last treatment, 3–4 fecal pellets from each mouse were freshly collected and placed in a sterile microcentrifuge tube, snap‐frozen in liquid nitrogen, and stored at −80 °C until use. Total genomic DNA was extracted using QIAamp fast DNA stool mini kit (Qiagen) following the manufacturer's instruction, and the DNA concentration and purity were determined by spectrophotometer (Eppendorf). The extracted DNAs were sent to the Beijing Genomics Institute (BGI TECH Solutions, Hong Kong) for meta 16S V3‐V4 region library preparation and paired‐end 300 bp (PE300) sequencing with at least 30K tags per sample, and subsequent bioinformatic analysis.

According to BGI, the protocols for library construction, sequencing, and bioinformation analysis are as follows: The qualified DNA templates (30 ng) were mixed with the 16S rRNA fusion primers and used for PCR amplication. The resulting PCR products were purified using Agencourt AMPure XP beads and utilized for library construction. Library size and concentration were assessed on an Agilent 2100 Bioanalyzer, and the qualified libraries were sequenced on a HiSeq platform according to the insert size. For bioinformatics analysis, raw data were filtered to remove adaptor pollution and low quality reads, after which clean pair‐end reads with overlap were merged to form tags and clustered into operational taxonomic units (OTU) by the scripts of the USEARCH software.^[^ ^48^ ^]^ Taxonomic classifications were assigned to OTU representative sequences using the Ribosomal Database Project database, and the analyses were performed based on OTU profile table and taxonomic annotation results.

### Statistical Analyses

All in vitro experiments were performed in replicates. Data are expressed as mean ± standard error of the mean (SEM). Statistical significance and the numbers of sample are noted in figures or legends where appropriate. Outliers are identified and removed using Tukey's fences. Statistical analyses were performed by one‐way analysis of variance (ANOVA) followed by the Tukey's post‐hoc test for multiple comparisons or unpaired two‐tailed Student's *t*‐test for comparison of two groups (GraphPad Prism 8.0). For all statistical tests, *P* <0.05 was considered statistically significant. (*P < 0.05, **P < 0.01, ***P < 0.001, ****P < 0.0001.)

## Conflict of Interest

The authors declare no conflict of interest.

## Supporting information

Supporting Information

## Data Availability

The data that support the findings of this study are available from the corresponding author upon reasonable request.
