# Supplementary material for: A Bioresponsive Genetically Encoded Antimicrobial Crystal for the Oral Treatment of Helicobacter Pylori Infection
Source: Adv Sci (Weinh). 2023 Sep 7;10(30):2301724. doi: 10.1002/advs.202301724 (PMC10602570; doi:10.1002/advs.202301724)
Supplement: Supplementary file 1 — Supporting Information [file ADVS-10-2301724-s001.pdf]

ADVANCED SCIENCE
Open Access

Supporting Information

for *Adv. Sci.*, DOI 10.1002/advs.202301724

A Bioresponsive Genetically Encoded Antimicrobial Crystal for the Oral Treatment of *Helicobacter Pylori* Infection

Wenxiu Zhang, Zaofeng Yang, Jiale Zheng, Kaili Fu, Jack Ho Wong, Yunbi Ni, Tzi Bun Ng, Chi Hin Cho, Michael K. Chan and Marianne M. Lee**

Supporting Information

A Bioresponsive Genetically Encoded Antimicrobial Crystal for the Oral Treatment of *Helicobacter pylori* Infection

Wenxiu Zhang, Zaofeng Yang, Jiale Zheng, Kaili Fu, Jack Ho Wong, Yunbi Ni, Tzi Bun Ng, Chi Hin Cho, Michael K. Chan, Marianne M. Lee**

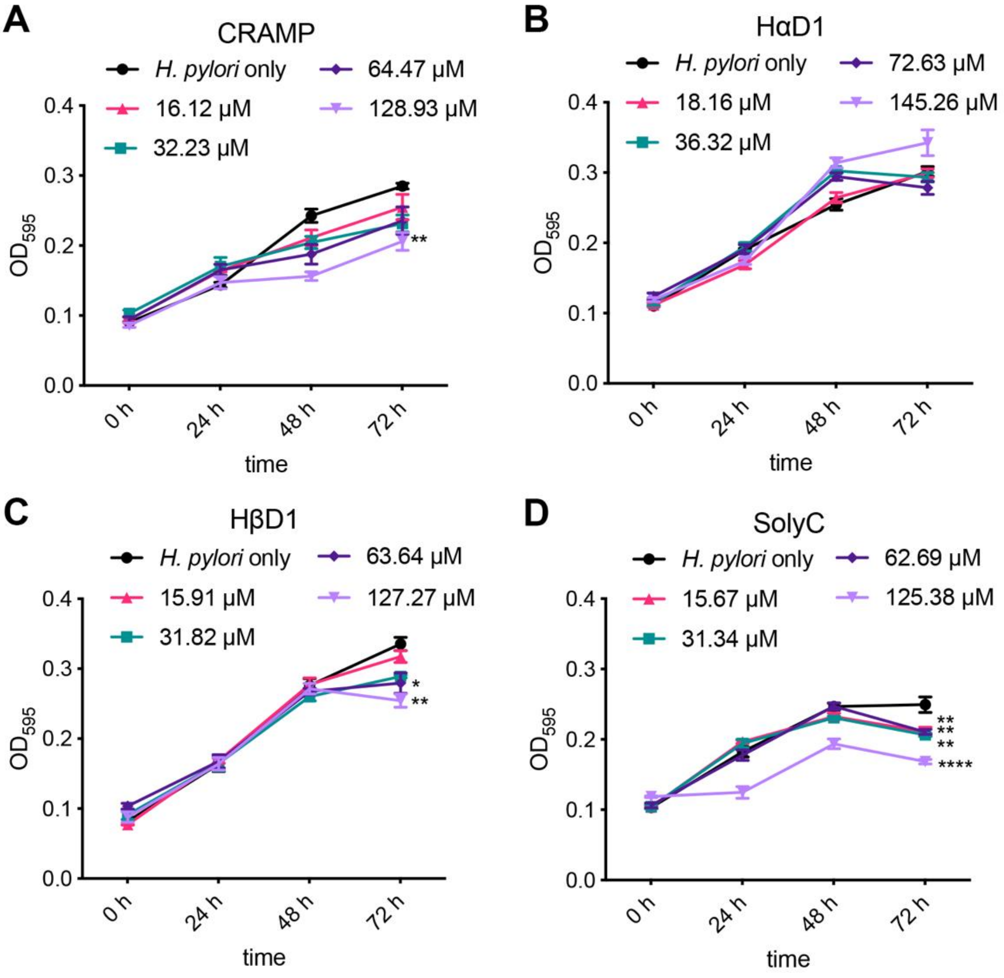

Figure S1. Antibacterial activity of antimicrobial peptides against *H. pylori* SS1 at different concentrations and time points. A) CRAMP, B) HαD1, C) HβD1, and D) SolyC. CRAMP, HβD1 and SolyC exhibited moderate bactericidal activity at high concentrations, while HαD1 exhibited no anti-*H. pylori* activity at the concentrations tested. **P* < 0.05, ***P* < 0.01, *****P* < 0.0001.

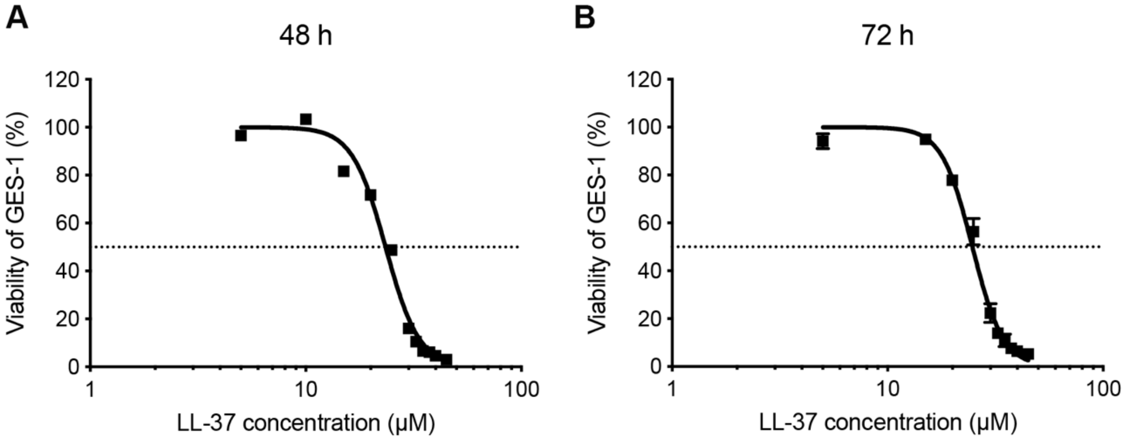

Figure S2. Cytotoxicity of LL-37 on human gastric epithelial cells GES-1. GES-1 cells were incubated with different concentrations, ranging from 5 - 45 μM, of LL-37 peptides for A) 48 h and B) 72 h. The IC_{50} was ~23.45 μM at 48 h and ~24.76 μM at 72 h.

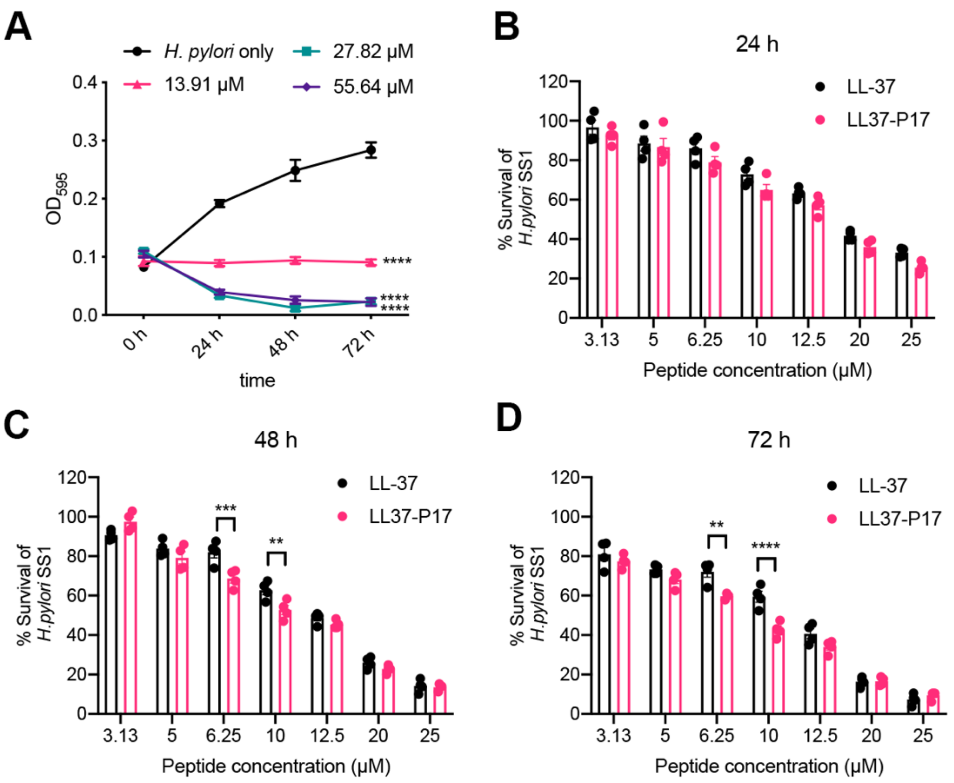

Figure S3. Effects of P17 conjugation on the antimicrobial activity of LL-37. A) The effect of LL37-P17 on the growth of *H. pylori* SS1 at different concentrations and time points. The MIC of LL37-P17 for *H. pylori* SS1 was ~13.91 μM. B-D) Comparison of anti-*H. pylori* activity of LL-37 and LL37-P17 peptides at B) 24 h, C) 48 h and D) 72 h. Data are represented as mean ± SEM. *$P < 0.05$, **$P < 0.01$, ***$P < 0.001$, ****$P < 0.0001$.

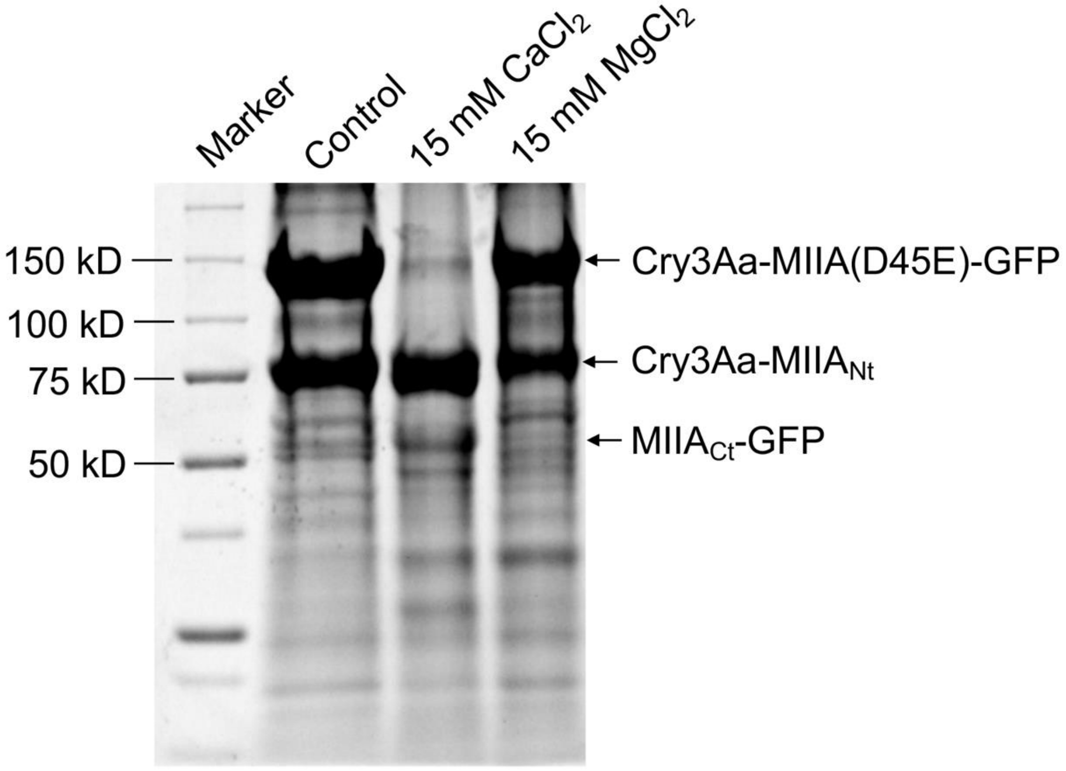

Figure S4. Cleavage of Cry3Aa-MIIA(D45E)-GFP was mediated by 15 mM $CaCl_2$, but not 15 mM $MgCl_2$.

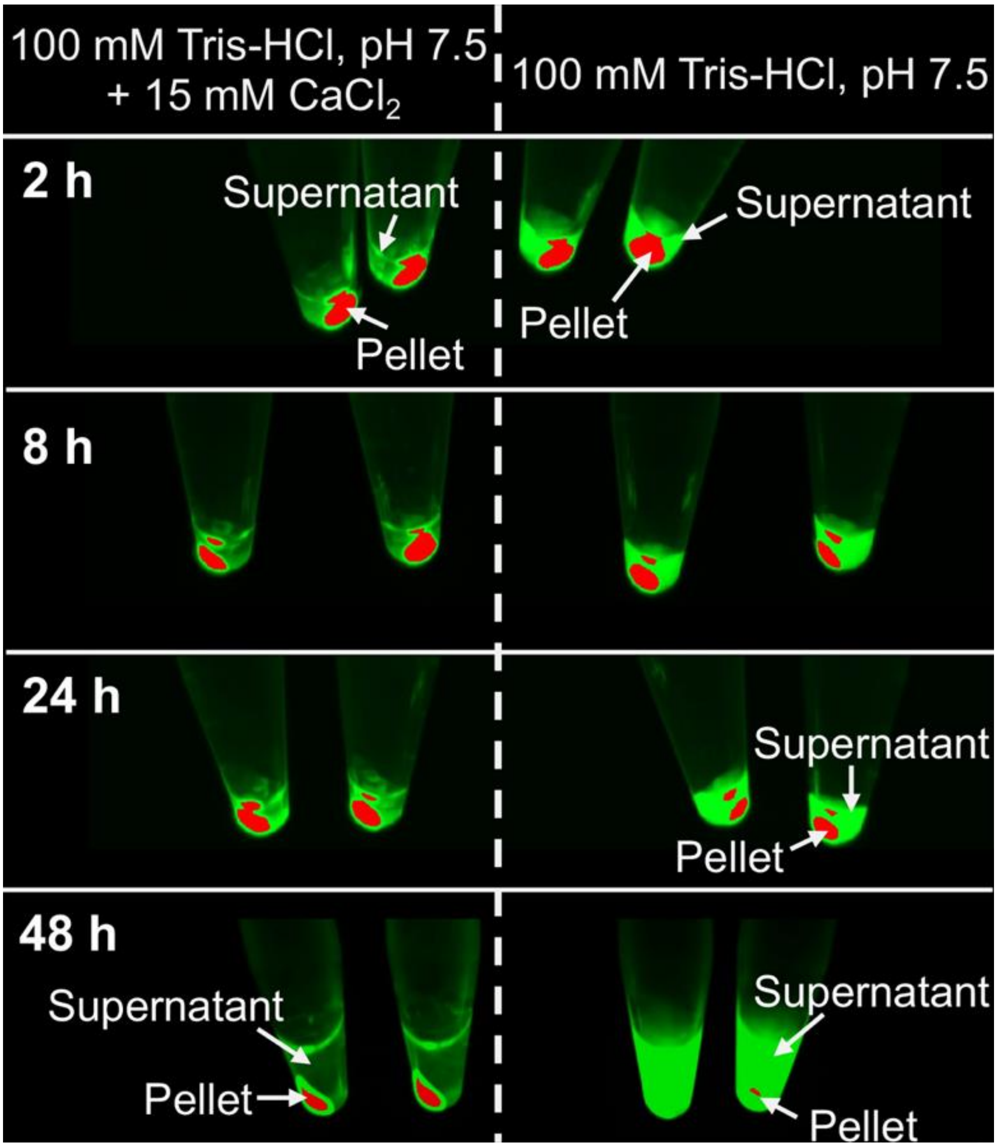

Figure S5. Release of $MIIA_{Ct}$-GFP from cleaved crystals in pH 7.5 buffer in the presence or absence of $CaCl_2$.

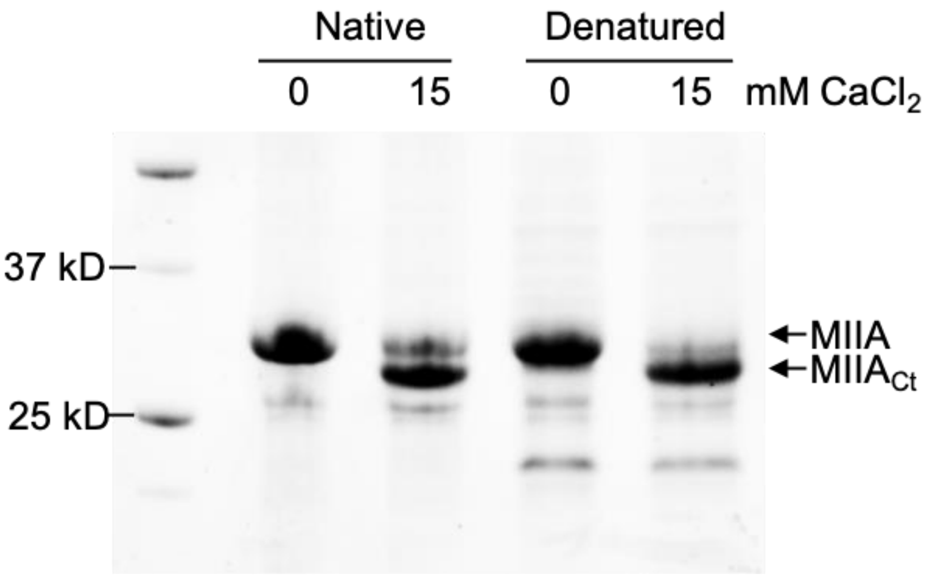

Figure S6. Native PAGE and denaturing SDS-PAGE analyses of wild type MIIA after induced cleavage by 15 mM $CaCl_2$.

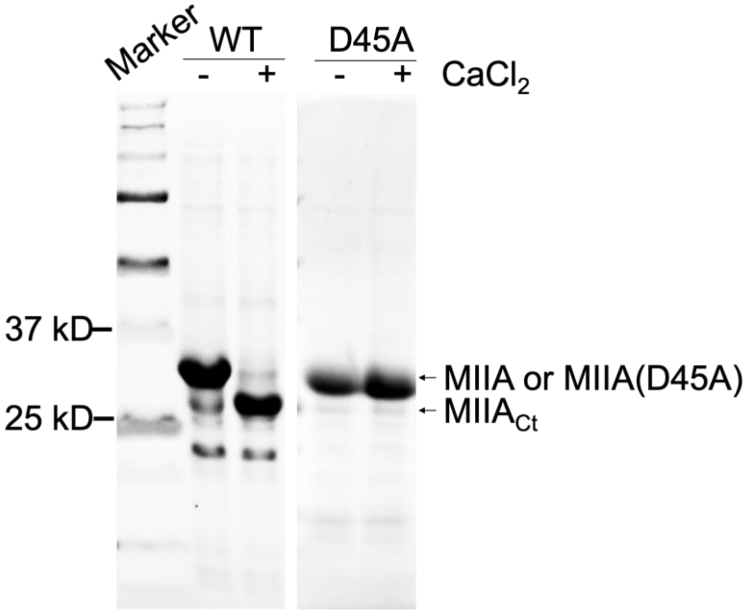

Figure S7. SDS-PAGE analysis showing that $CaCl_2$ induced the cleavage of wild type MIIA protein, but not MIIA(D45A) protein.

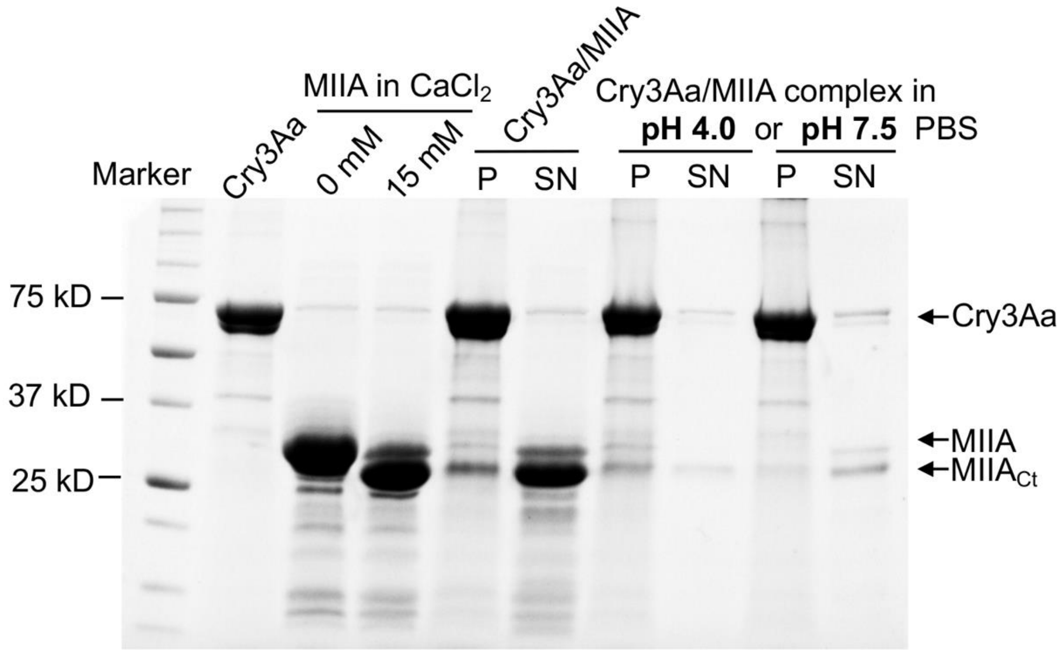

Figure S8. Original SDS-PAGE gel image of Figures 3E and 3F.

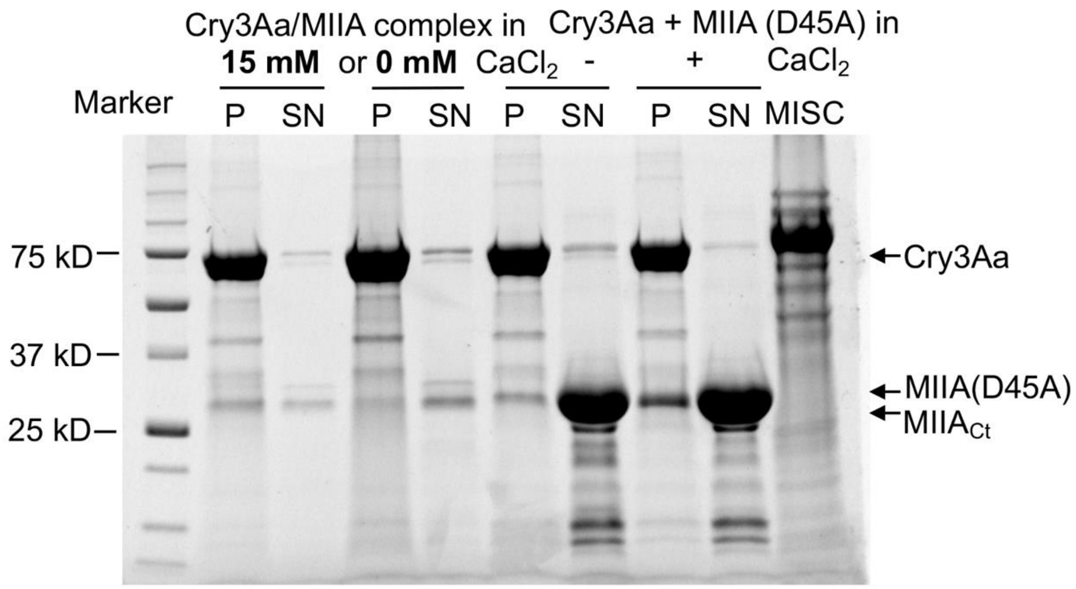

Figure S9. Original SDS-PAGE gel image of Figures 3G and 3H. MISC: Miscellaneous sample.

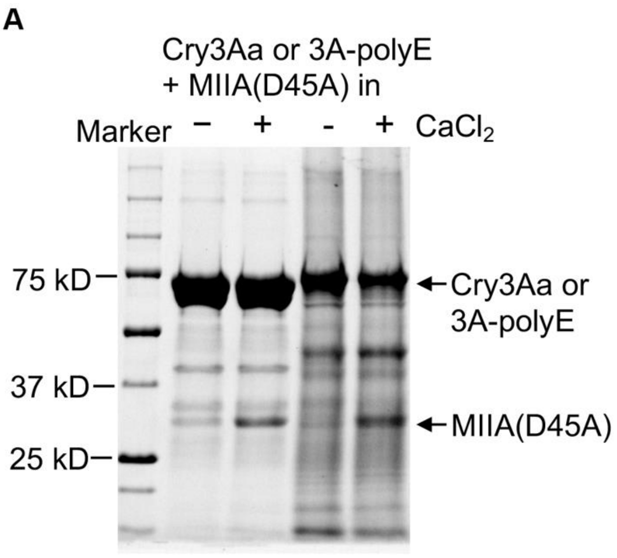

Figure S10. Binding of MIIA(D45A) protein to wild-type Cry3Aa and its negatively charged mutant in the presence of $CaCl_2$. A) SDS-PAGE analysis and B) bar plot showing that the negatively charged mutant of Cry3Aa (3A-polyE) crystals bind more MIIA(D45A) protein compared with wild-type Cry3Aa crystals in the presence of $CaCl_2$.

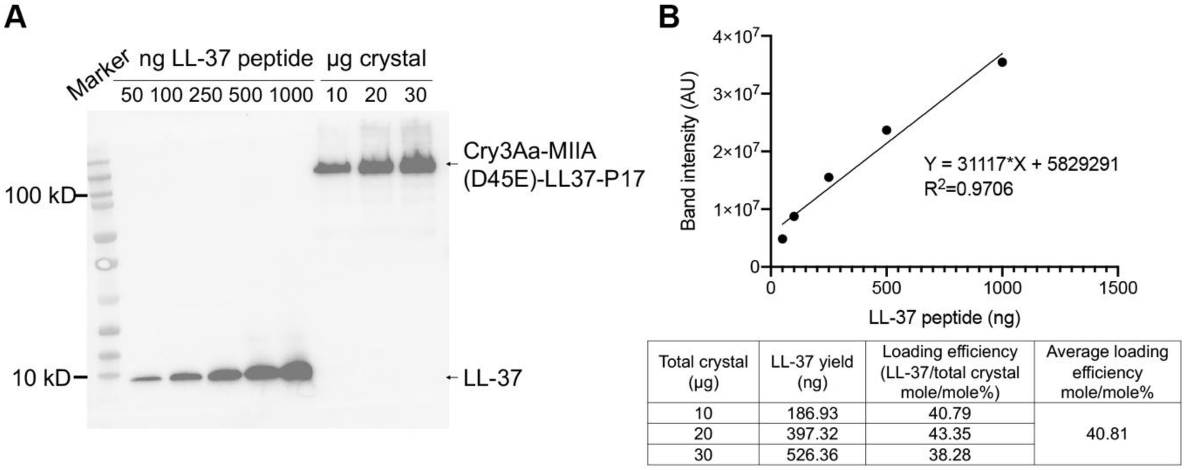

Total crystal (μg)	LL-37 yield (ng)	Loading efficiency (LL-37/total crystal mole/mole%)	Average loading efficiency mole/mole%
10	186.93	40.79	40.81
20	397.32	43.35	
30	526.36	38.28	

Figure S11. Quantification of LL-37 loaded in Cry3Aa-MIIA(D45E)-LL37-P17 crystals. A) Western blotting showing the increase in band intensity of LL-37 peptide and Cry3Aa-MIIA(D45E)-LL37-P17 crystal with ascending peptide/protein concentrations. B) The band intensities and the corresponding peptide concentrations of LL-37 peptide in (A) were used to derive the standard curve. Band intensity was measured by Bio-rad ImageLab software. Fit of the standard curve with simple linear regression model was performed using GraphPad Prism 8.0. Calculation of loading efficiency is based on band intensity of LL-37 in Cry3Aa-MIIA(D45E)-LL37-P17 crystals and the corresponding peptide concentration per the derived standard curve.

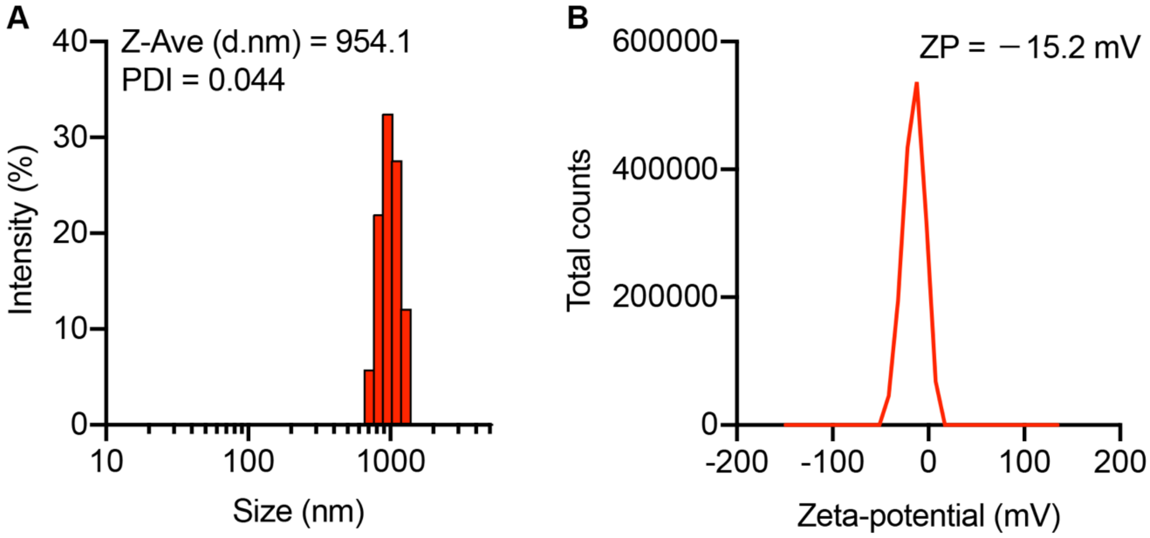

Figure S12. Characterization of activated Cry3Aa-MIIA(D45E)-LL37-P17 crystals. A) Size distribution and B) zeta-potential of activated Cry3Aa-MIIA(D45E)-LL37-P17 crystals.

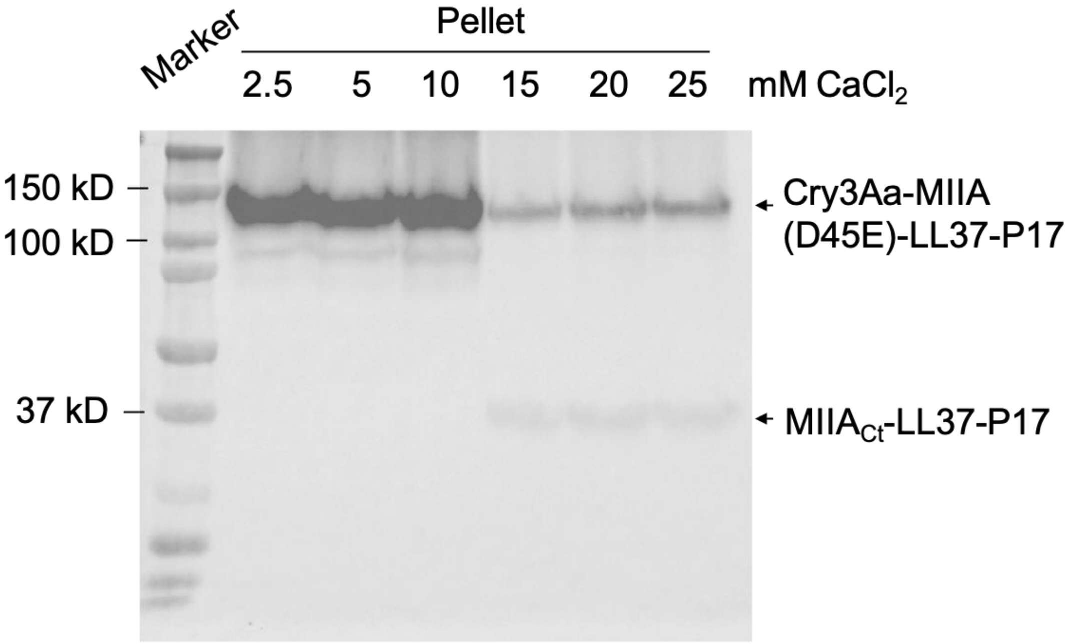

Figure S13. SDS-PAGE gel showing the cleavage efficiency of Cry3Aa-MIIA(D45E)-LL37-P17 crystals incubated in 2.5 - 25 mM $CaCl_2$ (100 mM Tris-HCl, pH 7.5) for 16 h. SDS-PAGE analysis indicated that 15 mM was the minimal Ca^{2+} concentration needed to achieve significant cleavage within the indicated length of time.

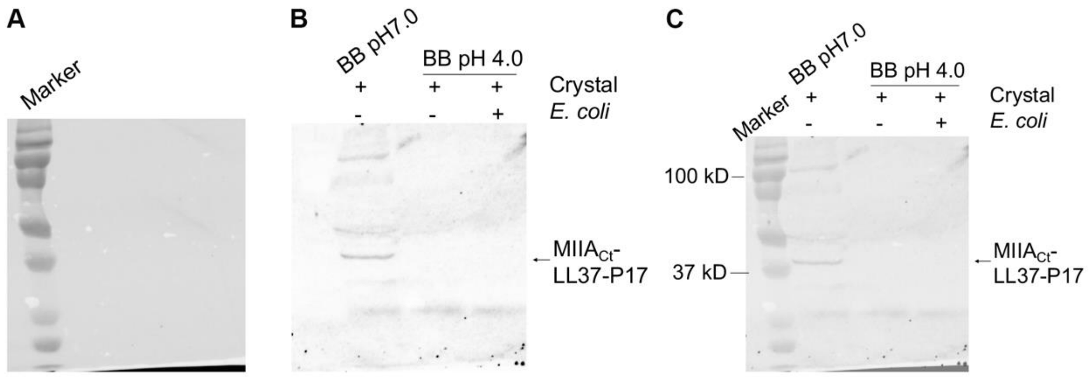

Figure S14. Original images of Figure 4G acquired using a BioRad ChemiDoc™ Touch Imaging System. The western blot membrane was imaged using A) the colorimetric channel to show the prestained molecular weight standards and B) the chemiluminescence channel to detect the protein samples. The two images were merged using the BioRad Imaging Software to produce C) the merged image, whose clarity is significantly poorer than that the spliced image depicted in Figure 4G.

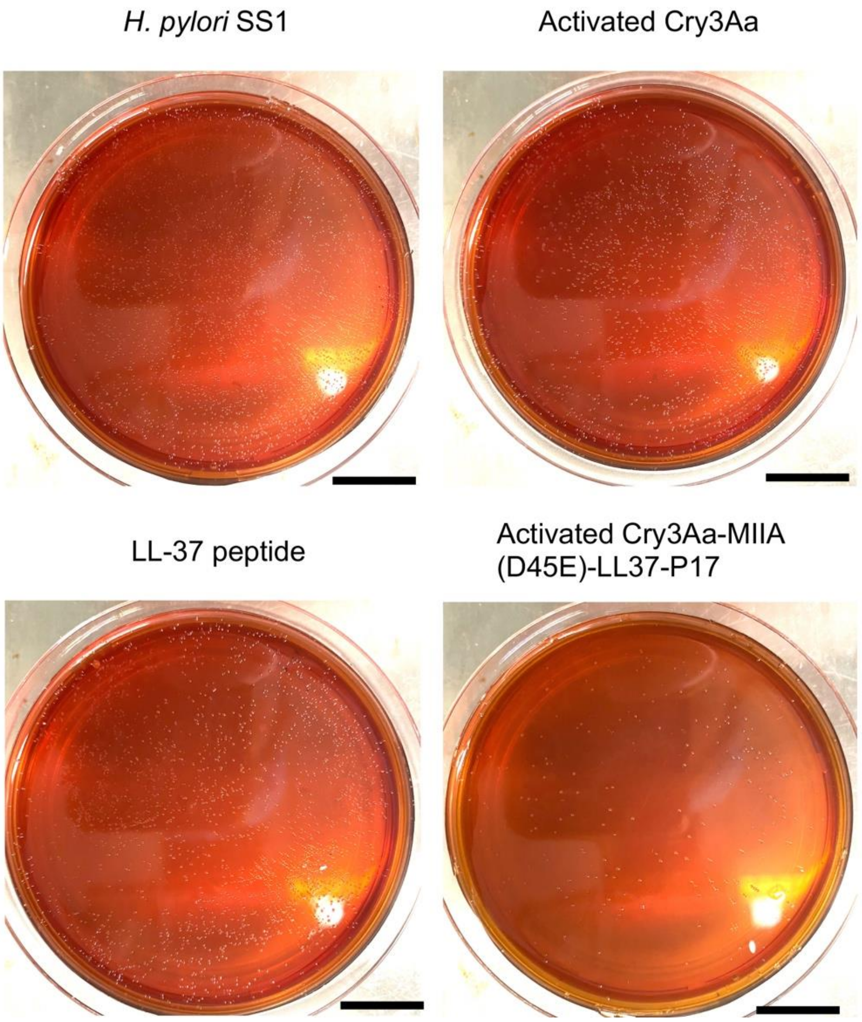

Figure S15. Images of *H. pylori* colony plates corresponding to Figure 4I. Scale bars: 2 cm.

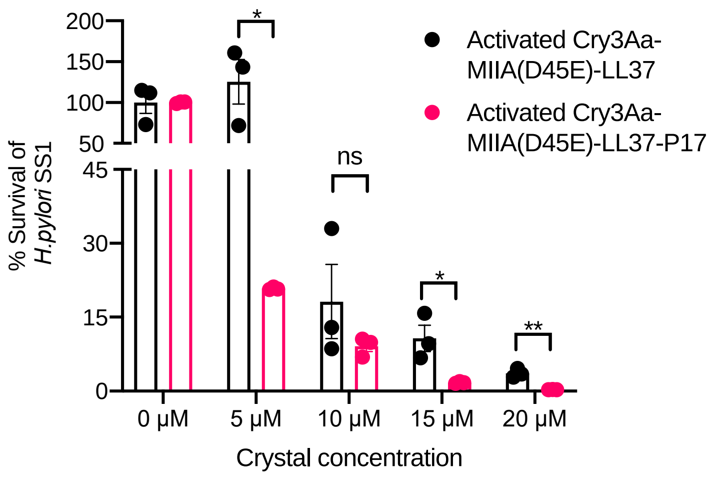

Figure S16. Effect of P17 conjugation to the antimicrobial activity of activated Cry3Aa-MIIA(D45E)-LL37-P17 crystals. *H. pylori* SS1 were treated with different concentrations of activated Cry3Aa-MIIA(D45E)-LL37 and Cry3Aa-MIIA(D45E)-LL37-P17 crystals for 24 h. Data are represented as mean ± SEM (N = 3). *P < 0.05, **P < 0.01. ns, not significant.

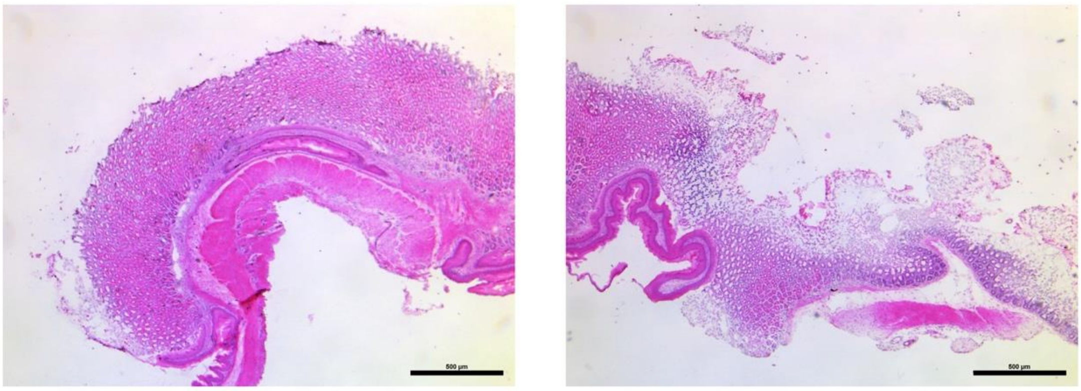

Figure S17. Corresponding H&E staining images for Figure 5F, upper and middle panels. Scale bars: 500 μm.

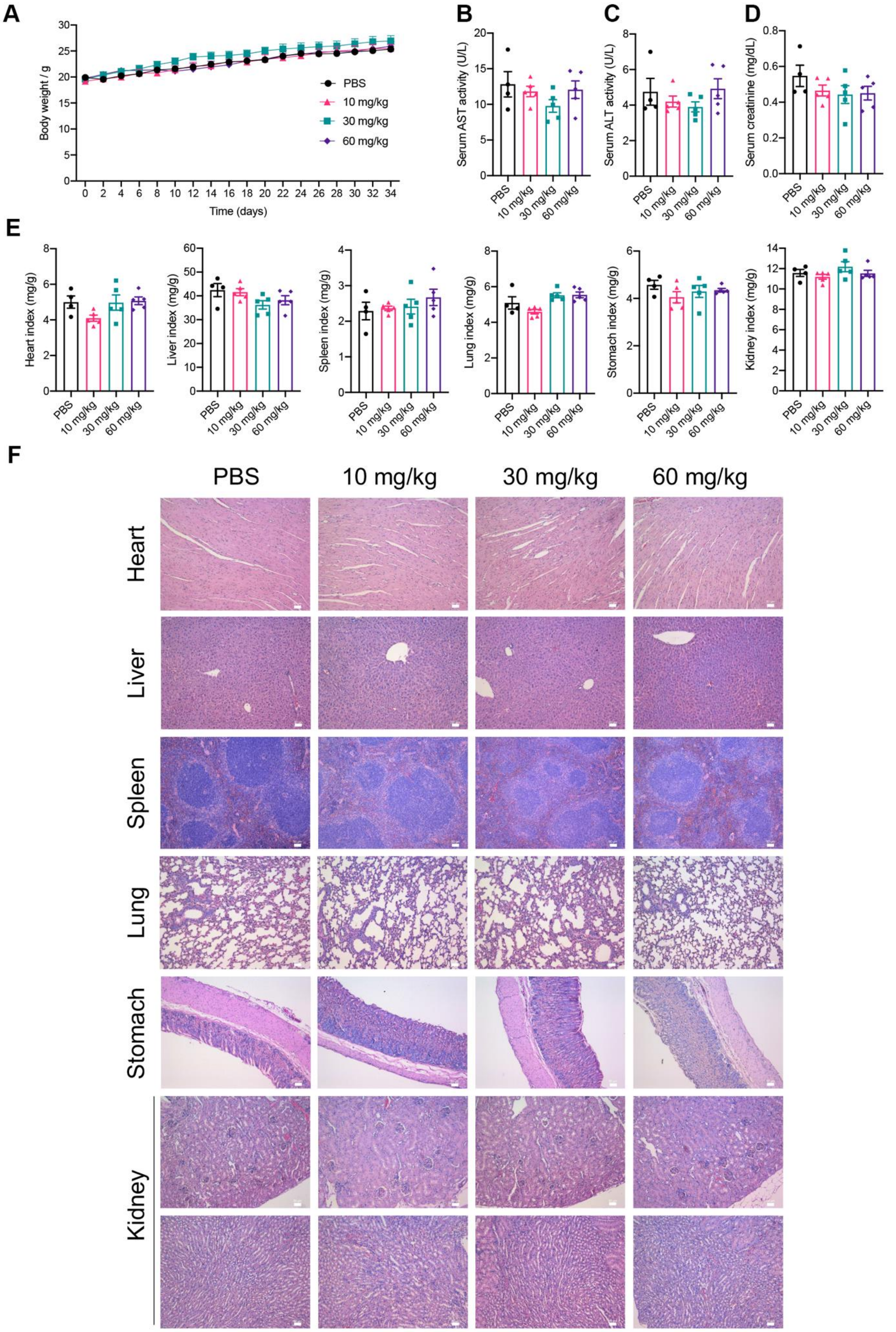
A
Body weight / g
Time (days)
PBS
10 mg/kg
30 mg/kg
60 mg/kg
B
Serum AST activity (U/L)
C
Serum ALT activity (U/L)
D
Serum creatinine (mg/dL)
E
Heart index (mg/g)
Liver index (mg/g)
Spleen index (mg/g)
Lung index (mg/g)
Stomach index (mg/g)
Kidney index (mg/g)
F
PBS
10 mg/kg
30 mg/kg
60 mg/kg
Heart
Liver
Spleen
Lung
Stomach
Kidney

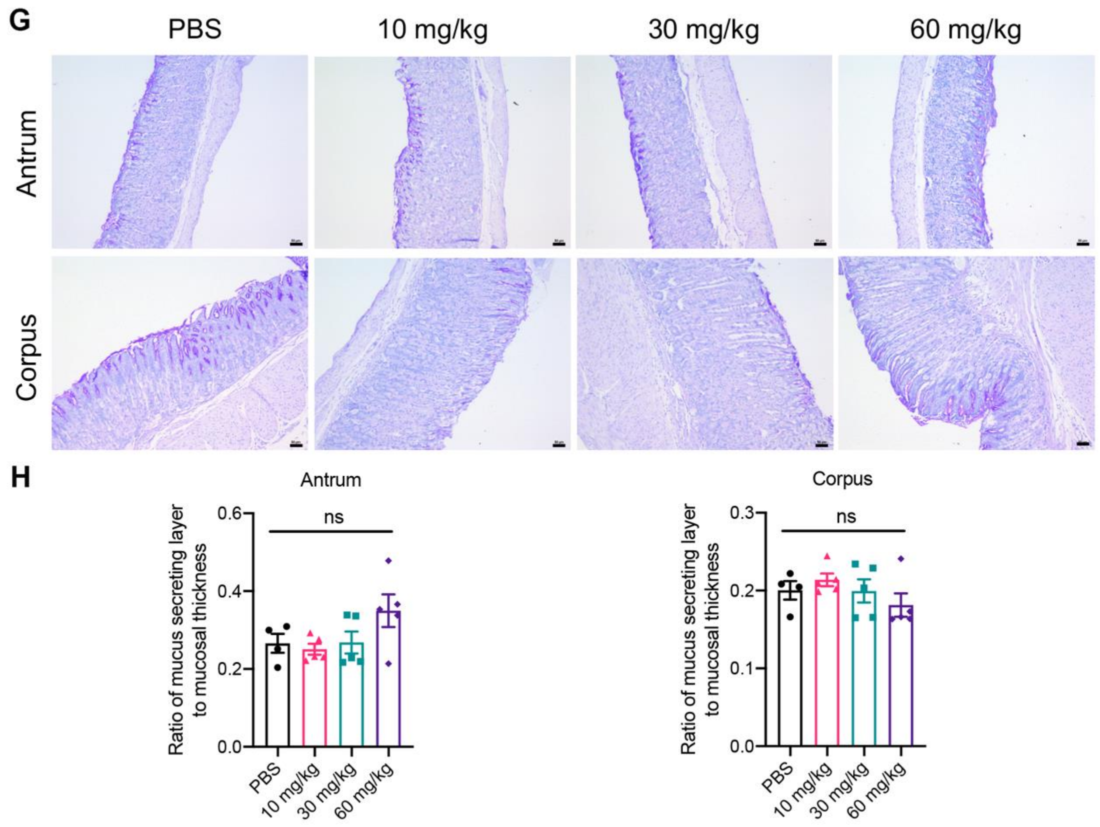

Figure S18. *In vivo* toxicity of activated Cry3Aa-MIIA(D45E)-LL37-P17 crystals. A) Body weights of C57BL/6 mice treated with different dosages of Cry3Aa-MIIA(D45E)-LL37-P17 crystals throughout the treatment period. Blood samples were collected at the end of treatment to measure the serum level of B) aspartate aminotransferase (AST), C) alanine aminotransferase (ALT) and D) creatinine. E) Corresponding organ index (organ weight/ body weight) including heart, liver, spleen, lung, stomach and kidneys of the mice in (A). Data are represented as mean ± SEM (*N* = 4 for PBS, *N* = 5 for other groups). F) Representative images of H&E-stained heart, liver, spleen, lung, stomach and kidney tissues showing no organ damage following repeated treatments with activated Cry3Aa-MIIA(D45E)-LL37-P17 crystals. Scale bars: 50 μm. G) Representative images of PAS-stained stomach antrum and corpus. Mucus cells were stained purple. Scale bars: 50 μm. H) Ratio of mucus secreting layer to mucosal thickness of both stomach antrum and corpus. For each mouse, more than 10 measurements from slides of two different portions of stomach tissues were examined.

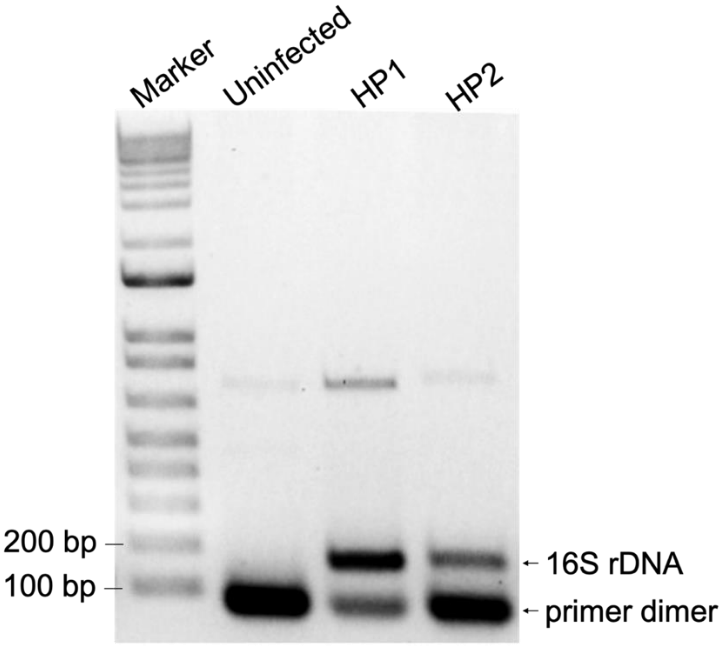

Figure S19. Agarose gel confirming *H. pylori* infection in mice. Genomic DNA was extracted from stomach tissues of mice oral gavaged with either BHI (uninfected control) or *H. pylori* (HP). The extracted DNA was used as template together with primers specific for *H. pylori* 16S rDNA for PCR amplification.

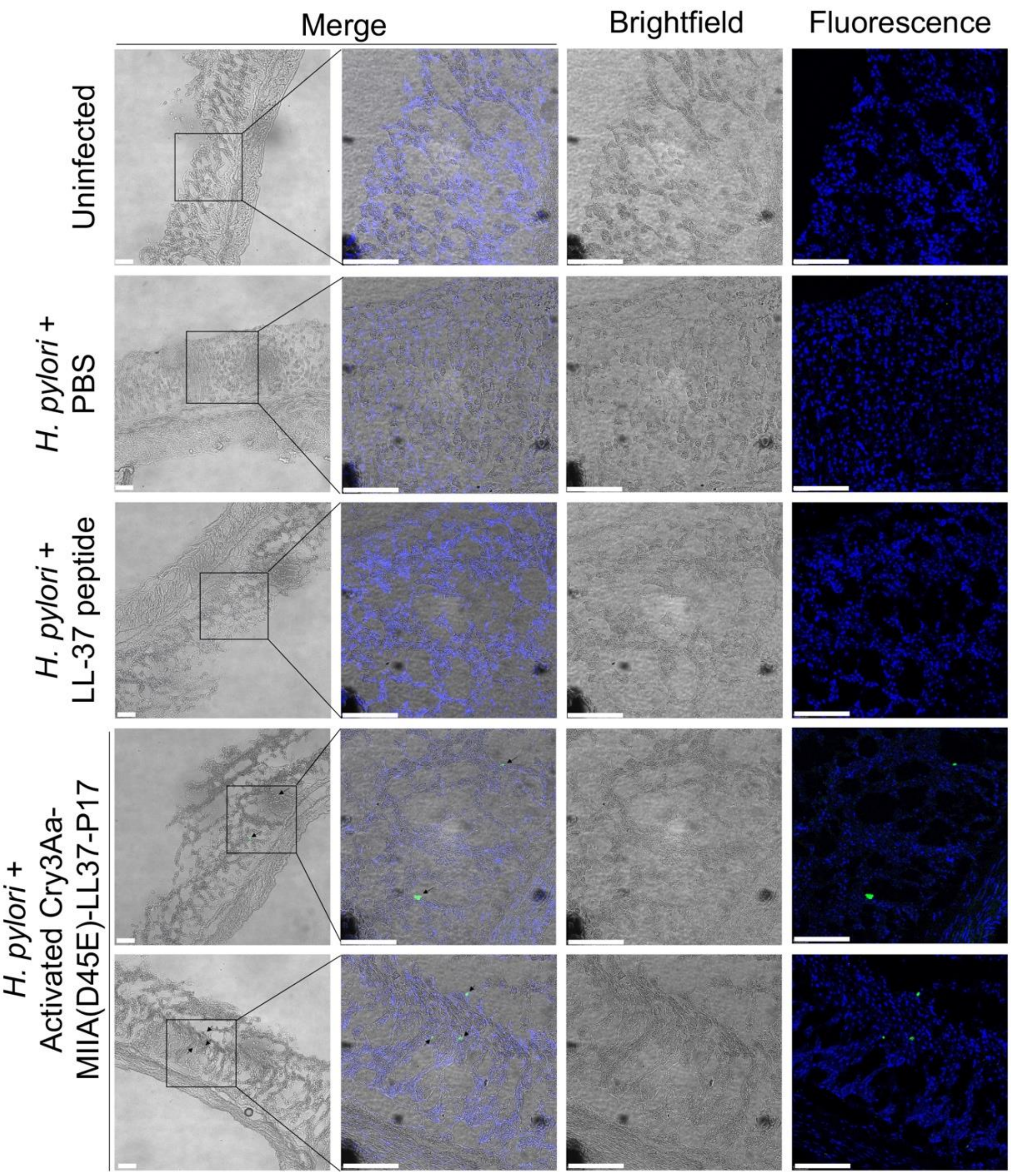

Figure S20. Immunofluorescence staining of LL-37 in mouse stomach. Sections were stained with anti-LL37 antibodies (Green) and Hoechst 33342 (Blue). Fluorescent and brightfield images were merged to show the architecture of the stomach tissue. The lowest panel displayed stomach section from the gastric corpus, while all other panels showed stomach sections from the antrum. Solid black arrows in the left panels indicate LL-37 in the gastric tissues merged image (leftmost panel) at 100x magnification, and (second left panel) at 400x magnification, corresponding to the green fluorescence signals shown in the fluorescence image at 400x magnification (rightmost panels). Scale bars: 100 μm.

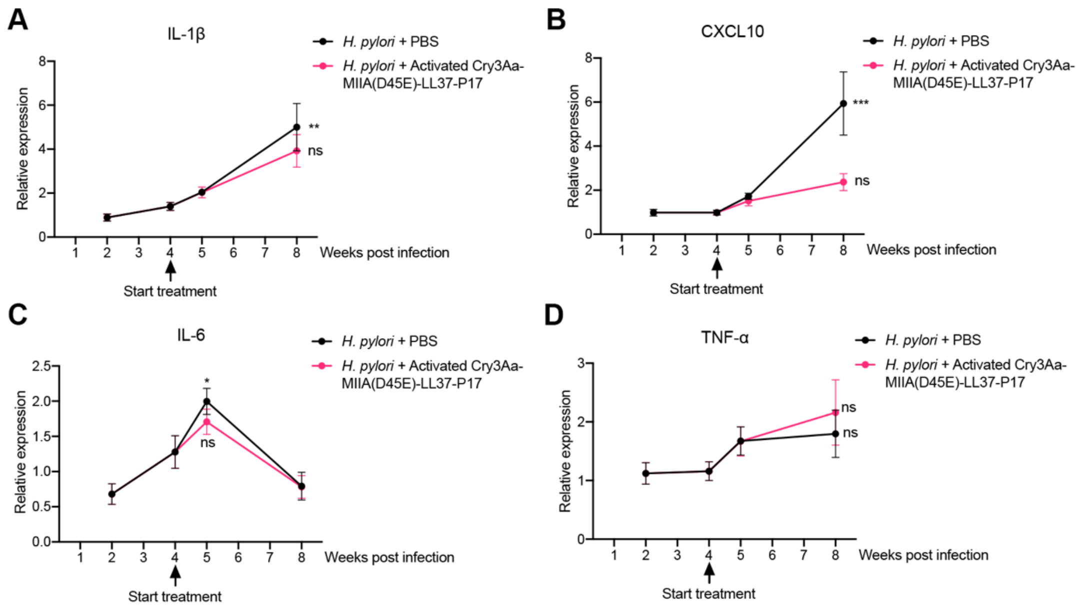

Figure S21. mRNA expression for cytokines A) IL-1β, B) CXCL10, C) IL-6 and D) TNF-α in mouse stomach tissues were quantitatively analyzed by real-time PCR. The cytokine mRNA expression was normalized against GAPDH. Data are represented as mean ± SEM ($N \geq 4$). Statistical analysis was tested against uninfected group, *$P < 0.05$, **$P < 0.01$, ***$P < 0.001$. ns, not significant.

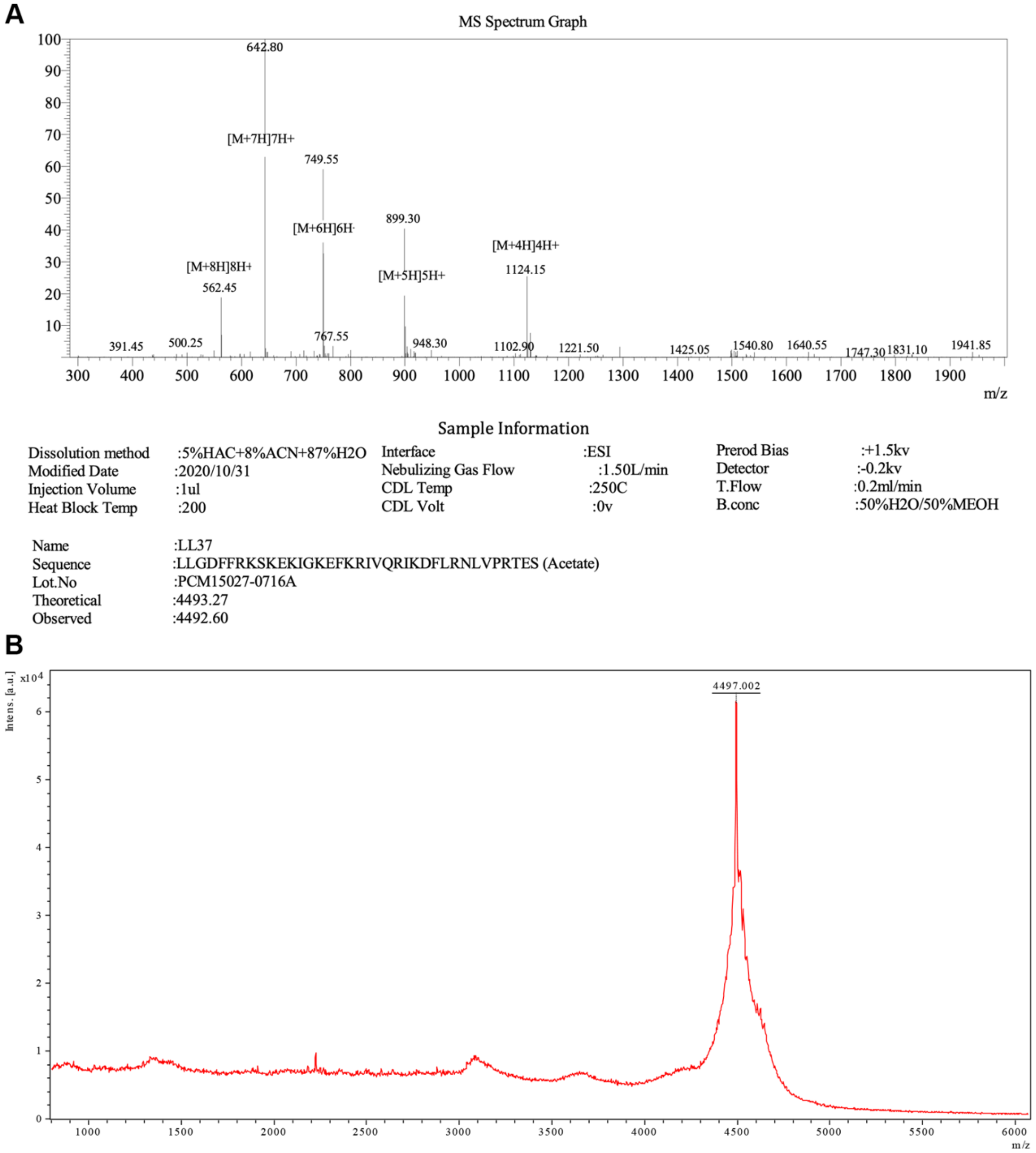

Figure S22. Mass spectra of LL-37 peptides. A) Mass spectrum of the LL-37 peptide provided by the peptide synthesis company Pepmic Co., Ltd. B) Mass spectrum of the synthesized LL-37 in (A) as verified by CUHK's in-house MALDI-TOF mass spectrometry.

Table S1. MICs of LL-37 peptide and clarithromycin against susceptible *H. pylori* SS1 and *H. pylori* recovered from treated mice infected with *H. pylori* SS1.

	Minimum inhibitory concentration (MIC)	
	LL-37 peptide (µM)	Clarithromycin (ng/mL)
H. pylori SS1	13.91 (1X)	62.5 (1X)
H. pylori recovered from treated-mice[a)]	13.91 (1X)	250 (4X)

[a)] *H. pylori* recovered from mice treated with activated Cry3Aa-MIIA(D45E)-LL37-P17 crystals were tested against LL-37 peptide, while colonies recovered from clarithromycin treated mice were tested against clarithromycin (*N* = 4).

Table S2. Relative abundance of species at phylum level in different groups.

Group	Uninfected	H. pylori + PBS	*H. pylori* + Activated Cry3Aa-MIIA(D45E)-LL37-P17	*H. pylori* + Clarithromycin
Deferribacteres (%)	0.00	0.00	0.04±0.01	0.03±0.01
Candidatus_Saccharibacteria (%)	0.31±0.05	0.31±0.04	0.25±0.05	0.21+0.04
Verrucomicrobia (%)	0.31±0.11	0.00	0.40±0.19	0.82±0.44
Other (%)	0.47±0.08	0.17±0.10	0.89±0.21	0.98±0.51
Actinobacteria (%)	1.32±0.48	0.11±0.01	0.63±0.12	2.68±1.06
Tenericutes (%)	2.07±1.12	0.00	2.94±1.28	1.22±0.34
Proteobacteria (%)	5.43±1.49	2.89±0.74	6.66±1.89	3.60±0.59
Firmicutes (%)	52.41±3.19	34.77±2.57 ****	48.00±3.92	40.36±6.30 **
Bacteroidetes (%)	36.00±4.76	61.92±3.32 ****	40.63±6.13	50.81±6.18 ***

Statistical tests were compared with uninfected group. Data are represented as mean ± SEM. $^{**}P < 0.01$, $^{***}P < 0.001$, $^{****}P < 0.0001$.

Table S3. Amino acid sequences of the synthesized antimicrobial peptides.

Peptide	Sequence
LL-37	LLGDFFRKSKEKIGKEFKRIVQRIKDFLRNLVPRTES
LL37-P17	LLGDFFRKSKEKIGKEFKRIVQRIKDFLRNLVPRTES GKPLGNN
CRAMP	GLLRKGGEKIGEKLKKIGQKIKNFFQKLVPQPEQ
HαD1	ACYCRIPACIAGERRYGTCIYQGRLWAFCC[a]
HβD1	DHYNCVSSGGQCLYSACPIFTKIQGTCYRGKAKCCK[b]
SolyC	FSGGNCRGFRRRCFCTK-NH_2

[a)] Disulphide bridges at C2-C30, C4-C19, C9-C29; [b)] Disulphide bridges at C5-C34, C12-C27 and C17-C35.

Table S4. Amino acid sequences of the three 7-mer *H. pylori*-binding peptides.

Peptide	Sequence
P7	LSDDTSN
P8	SEAFADI
P17	GKPLGNN

Table S5. Primers used in real-time PCR.

Gene name	Sequence
GAPDH	Forward: 5'- GCAGTGGCAAAGTGGAGATT -3'
	Reverse: 5'- TCT CCATGGTGGTGAAGACA -3'
CXCL10	Forward: 5'- GGATGGCTGTCCTAGCTCTG -3'
	Reverse: 5'- ATAACCCCTTGGGAAGATGG -3'
IL-1β	Forward: 5'- TCAGGCAGGCAGTATCACTCA -3'
	Reverse: 5'- GGAAGGTCCACGGGAAAGA -3'
IL-6	Forward: 5'- ACAACCACGGCCTTCCCTACTT -3'
	Reverse: 5'- GTGTAATTAAGCCTCCGACT -3'
TNF-α	Forward: 5'- CGTGCTCCTCACCCACAC -3'
	Reverse: 5'- GGGTTCATACCAGGGTTTGA -3'
